# Global dynamic optimization approach to predict activation in metabolic pathways

**DOI:** 10.1186/1752-0509-8-1

**Published:** 2014-01-06

**Authors:** Gundián M de Hijas-Liste, Edda Klipp, Eva Balsa-Canto, Julio R Banga

**Affiliations:** 1 Bioprocess Engineering Group, Spanish National Research Council, IIM-CSIC, C/Eduardo Cabello 6, 36208 Vigo, Spain; 2 Theoretical Biophysics, Humboldt-Universität zu Berlin, Invalidenstr. 42, 10115 Berlin, Germany

**Keywords:** Dynamic optimization, Global optimization, Multi-objective optimization, Pareto optimality, Metabolic pathways, Gene expression

## Abstract

**Background:**

During the last decade, a number of authors have shown that the genetic regulation of metabolic networks may follow optimality principles. Optimal control theory has been succesfully used to compute optimal enzyme profiles considering simple metabolic pathways. However, applying this optimal control framework to more general networks (e.g. branched networks, or networks incorporating enzyme production dynamics) yields problems that are analytically intractable and/or numerically very challenging. Further, these previous studies have only considered a single-objective framework.

**Results:**

In this work we consider a more general multi-objective formulation and we present solutions based on recent developments in global dynamic optimization techniques. We illustrate the performance and capabilities of these techniques considering two sets of problems. First, we consider a set of single-objective examples of increasing complexity taken from the recent literature. We analyze the multimodal character of the associated non linear optimization problems, and we also evaluate different global optimization approaches in terms of numerical robustness, efficiency and scalability. Second, we consider generalized multi-objective formulations for several examples, and we show how this framework results in more biologically meaningful results.

**Conclusions:**

The proposed strategy was used to solve a set of single-objective case studies related to unbranched and branched metabolic networks of different levels of complexity. All problems were successfully solved in reasonable computation times with our global dynamic optimization approach, reaching solutions which were comparable or better than those reported in previous literature. Further, we considered, for the first time, multi-objective formulations, illustrating how activation in metabolic pathways can be explained in terms of the best trade-offs between conflicting objectives. This new methodology can be applied to metabolic networks with arbitrary topologies, non-linear dynamics and constraints.

## Background

Optimality principles have been successfully used to describe the design, organization and behavior of biological systems at different levels. Sutherland [1] argues that optimization can in fact play an even major role, allowing biology to move from just describing mechanisms to being able to predict, from first principles, how organisms should be designed. In the context of cell biology, mathematical optimization has been the underlying hypothesis in applications such as flux balance analysis [2], the analysis of activation of metabolic pathways [3-7], model identification (including parameter estimation and optimal experimental design) [8] and optimal stimulation procedures to achieve a desired biological behavior [9-11].

Here we focus on the problem of enzyme activation in metabolic networks, which has received substantial attention in the recent literature. Several authors have shown that the genetic regulation of metabolic networks may follow an optimality principle, such as the minimization of the transition time or the maximization of the production of a given metabolite. In their seminal work, Klipp et al. [4] showed how sequential gene expression appears in unbranched metabolic networks under the hypothesis of minimum transition time. The sequence matched the enzyme order in the pathway. This *“just-in-time”* activation pattern was experimentally confirmed later for the case of the amino-acid biosynthesis systems of *E. coli*[5]. These authors also showed that in the arginine, methionine and serine systems, the earlier the enzyme is involved in a pathway, the shorter is the response time and the higher the maximal promoter activity of the corresponding gene. Evidences of temporal distribution of enzymes were also found in the lysine biosynthesis pathway by *E. coli*[12]. Chechik et al. [13] proposed the notion of activity motif to systematically study the dynamic behavior of metabolic networks. As a case study they considered the transcriptional response of metabolic genes after a sudden change in environmental or nutritional condition in *S. cerevisiae*. These authors also found that enzymes in a metabolic chain are induced in the same order they are used in the pathway in both directions forward and backward. All these results support the idea that *“just-in-time”* activation in metabolic pathways is a widespread phenomena.

These works indicate that, starting with a mathematical model of a given metabolic network, it is in principle possible to anticipate activation profiles for specific cellular objectives solving the corresponding dynamic optimization problem. Needless to say, the results obtained must be compared with existing or new experimental measurements. In this dynamic optimization framework, the objective is to compute time varying control profiles, usually enzyme concentrations or the corresponding expression rates, so as to minimize a given cost function, such as the time needed to reach a given amount of product, subject to the system dynamics (the model) and algebraic constraints, for example in the maximum amount of enzyme available.

However solving this class of optimization problems for arbitrary metabolic networks of certain complexity is still a challenge. So far, the literature reports applications in very simple scenarios. Oyarzún et al. [6,14] suggested the application of classical optimal control theory, giving an analytical proof of sequential activation for unbranched networks for the case the transition time is to be minimized subject to specific constraints. Almost in parallel, Bart et al. [7,15] also considered the maximum principle of Pontryagin to solve related examples. Although successful for simple unbranched pathways, these works suggest that the use of indirects approaches from optimal control theory, such as the maximum principle of Pontryagin or the use of the Hamilton-Jacobi-Bellman equation, may not be appropriate to deal with general problems (such as those related to branched and/or large networks) due to the complexity of the resulting numerical problems.

It is also important to highlight that all these authors have studied enzyme activation in metabolic networks considering that the metabolism is optimal with respect to a single-objective. We believe that a more realistic approach is to take more than one objective into account. Multi-objective optimization has been successfully applied in several classes of biological problems [16], including metabolic engineering [17,18], the heat-shock response of bacteria [19], and the allosteric regulation of enzymes [20]. These and other recent works [21-23] illustrate the importance of studying optimality as the trade-off between conflicting objectives, such as economy (cost) and effectiveness (benefit), in order to obtain results with more biological meaning. It should be noted that multi-objective optimization problems do not have a unique solution, but a set of optimal solutions known as the Pareto front. All Pareto solutions are optimal but represent different trade-offs between objectives.

Here we consider, for the first time, a multi-objective formulation to explain activation in metabolic networks. It should be noted that several authors [6,14,24] have considered composed objective functions, such us combinations of transition time and enzyme consumption, thus obtaining only one of the possible Pareto solutions instead of a Pareto front. In this work we adopt a general multi-criteria framework and we propose the use of advanced numerical dynamic optimization techniques to study/predict enzyme activation in general pathways. The underlying idea is to combine the control vector parameterization (CVP) approach with adequate global optimization techniques. This new methodology can be applied to metabolic networks with arbitrary topologies, non- linear dynamics and constraints.

We then illustrate the approach with two sets of problems. First, a set of single-objective examples of increasing complexity taken from the literature. The multimodality of these problems is evaluated by means of multi-start local deterministic methods. The need of global optimization methods arises from the non-convexity of the general problem, due to the frequent bi-linear terms for the controls, the non-linear character of the systems dynamics, and the presence of (nonlinear) constraints [25]. As a consequence, the use of standard local optimization methods results in local solutions. To surmount these difficulties, we also present a metaheuristic approach which is compared with several other stochastic global optimization methods. Second, we consider the multi-objective formulation of several problems, illustrating how activation in metabolic pathways can be explained in terms of the best trade-offs between conflicting objectives.

## Methods

### Problem formulation

The aim of the general *multi-objective dynamic optimization problem* is to compute the time varying control profiles and the final time (**u**(*t*), *t*_*f*_) so as to minimize (or maximize) a given set of cost functions (**J**) subject to the system dynamics and possibly algebraic constraints. Mathematically this reads:

(1) $$\underset{u(t),t_{f}}{\min}J\left[x,u\right]$$

Where:

$$J\left[x,u\right]=\left[\begin{array}{c} J_{1}\left[x,u\right]\\ J_{2}\left[x,u\right]\\ \vdots \\ J_{n}\left[x,u\right] \end{array}\right]$$

Subject to:

(2) $$\frac{dx}{\text{dt}}=\tilde{\Psi }\left[x\{t\},u\{t\},t\right],x\left(t_{0}\right)=x_{0}$$

(3) $$\begin{array}{lcr} & & h[\;x(t),u(t)]=0 \end{array}$$

(4) $$\begin{array}{lcr} & & g[\;x(t),u(t)]\leq 0 \end{array}$$

(5) $$\begin{array}{lcr} & & \mathrm{ph}[\;x(t_{i}),u(t_{i})]=0 \end{array}$$

(6) $$\begin{array}{lcr} & & \mathrm{pg}[\;x(t_{i}),u(t_{i})]\leq 0 \end{array}$$

(7) $$\begin{array}{lcr} & & u^{L}\leq u(t)\leq u^{U} \end{array}$$

where: 

•The costs functional (1) corresponds, for example, to the time needed to reach a given state of the system or the enzyme cost. In a single-objective problem *J*[**x**,**u**]=*J*_1_[**x**,**u**];

•**x** is the vector of state variables, typically metabolite concentrations;

•**u** is the vector of control variables: enzyme concentrations (**e**) or expression rates (**r**);

•Equation (2) represents the system dynamics (dynamic mathematical model of the network);

•Equations (3)–(4) represent equality and inequality path constraints, such as the total amount of enzyme available during the process;

•Equations (5)–(6) represent equality and inequality point constraints, i.e. those that must be verified at a given time, for example the amount of metabolite at the end of the process;

•Equation (7) corresponds to the upper and lower bounds for the control variables, for instance the minimum and maximum enzyme available through out the process.

### Numerical methods

Roughly speaking, numerical methods for the solution of dynamic optimization problems can be classified into two groups: direct and indirect methods. Indirect methods solve the optimization problem using the Pontryagin’s maximum principle. This method is based on the transformation of the original problem using the necessary optimality conditions of Pontryagin. This results in a two or multi-point boundary value problem, in the presence the constraints, to be solved for state and co-state variables.

Direct approaches, such us complete parameterization (CP, [26]), multiple shooting (MS, [27]) or control vector parameterization (CVP, [28]) transform the original problem into a non-linear programming problem (NLP) by means of the discretization and approximation, either of the control variables or both the control and state variables. The main differences among these methods are the number of decision variables, the presence or absence of parameterization constraints and the necessity of using an initial value problem solver. The use of CP or MS results in larger NLP problems with junction constraints to handle the system dynamics, which requires the use of specific optimization methods and may be computationally intensive for large scale dynamic systems. Therefore the CVP method is selected here.

#### Control vector parametrization

In the CVP approach the time horizon is divided into a number of *ρ* time intervals, with variable or fixed length. The control variables are then approximated within each interval by means of low order polynomials. With this parametrization the original dynamic optimization problem is transformed into a non-linear programming problem with dynamic and algebraic constraints. The non-linear programming problem obtained must be solved by employing a suitable NLP method and an initial value problem solver.

#### Nonlinear programming methods

Nonlinear programming methods are basically classified in local and global methods. Local methods are designed to converge to the closest solution to the initial guess. The iterates are computed by means of the cost function value or the gradient and/or the Hessian of the cost function with respect to the decision variables. In addition there are implementations that are able to automatically handle constraints. In the context of dynamic optimization, Sequential Quadratic Programming (SQP) methods, which guarantee feasibility of the solution at convergence, or feasible SQP methods, which guarantee feasibility throughout the optimization, have shown to be efficient in the solution of large-scale constrained NLPs.

Unfortunately, NLPs arising from the application of direct approaches (such as CVP) are frequently multimodal. This is particularly the case in the presence of non-linear or bi-linear dynamic constraints (as in the problems considered in this work) or when variable length elements are used in the CVP approach. Therefore, local optimization techniques may converge to local optima. A multi-start of local methods may help to analyze multimodality of the optimization problem. Interested readers will find illustrative examples in the Additional file 1.

Stochastic global optimization methods are robust alternatives for the search of the global solution. However these methods are well known to be particularly inefficient in the refining of the solutions. In this concern, Balsa-Canto et al. [29], presented the combination of a stochastic global solver with a deterministic local method in a so called sequential hybrid approach for the solution of dynamic optimization problems. That work highlights the role of the tuning of the hybrid, i.e. the selection of the methods and the switching point, in the overall performance of the method. However several examples illustrated the benefits of the combination with respect to the individual solvers in the sense of robustness and efficiency.

Recent works [30,31] show how the scatter search metaheuristic in combination with local methods outperforms previous sequential hybrid methods in the solution of complex optimization problems. The key property of this approach is that the switching points are automatically selected by the algorithm based on a balance between quality and diversity among the solutions generated in every iteration.

Based on the above, the following methods were selected attending to their previously reported performance: 

•SRES, the stochastic ranking evolution strategy method [32]. An evolutionary approach that is able to handle constraints by means of the stochastic ranking approach.

•DE, differential evolution [33]. It is based on the generation of new solutions by adding the weighted difference between two population vectors to a third vector. The method was complemented with a death penalty function approach to handle constraints.

•Sequential hybrids of SRES or DE with different implementations of successive quadratic programming methods.

•eSS, scatter search [34] in combination with different implementations of successive quadratic programming methods.

#### Multi-objective optimization methods

In contrast to the single-objective case, the aim in a multi-objective case is to find the optimal trade-offs between conflicting objectives. The notion of a single optimal solution is replaced by the notion of a Pareto front, i.e. a set of optimal trade-off solutions. All solutions in this set are optimal in the sense that it is not possible to improve one of the objectives without degrading one or more of the others. The weighted sum or the *ε*−constraint approach transforms the multi-objective problem into a set of single-objectives problems whose solutions result in the Pareto front. However the use of the weighted sum presents a number of drawbacks such as the impossibility to recover non-convex area in the Pareto front or the selection of weigths to obtain a uniform distribution of Pareto solutions. In view of this, the *ε*−constraint method [18] was selected here. The underlying idea is to solve a set of single-objective problems where one objective (*J*_*p*_) is to be minimized while the others (*J*_*i*_) are incorporated as inequality constraints. Mathematically this reads as:

(8) $$\underset{u(t),t_{f}}{\min}J_{p}\left[x,u\right]$$

Subject to:

(9) $$J_{i}\left[x,u\right]\leq \varepsilon_{i}\;i=1,\mathrm{...},n\;\text{and}\;i\neq p$$

The Pareto front is obtained modifying the upper-bounds of the constraints. Each of the single-objective problems was solved using control vector parametrization combined with the eSS scatter search solver.

## Results and discussion

This work considers the solution of two sets of examples. The first set consists of single-objective problems taken from the literature. All these problems were initially solved with a multi-start of local methods to analyze their possible multimodal nature. Since all problems appeared to be multimodal, a selection of global and hybrid methods were then used to find the global solution for each case study. The details are listed in Additional file 1, showing that scatter search (eSS) offered the best compromise between efficiency and robustness. A second set of problems considered a more general multi-objective formulation. The *ε*−constraint approach and scatter search (eSS) were subsequently used to solve this second set of problems, enabling us to obtain the optimal trade-offs between different objectives. A detailed analysis of the resulting enzyme activation profiles revealed the *“just-in-time”* activation is still present in multi-criteria optimality. The examples considered are summarized in Table 1. Results are presented and discussed below.

**Table 1:** Summary of the pathways considered together with the objectives used for each example

**Label**	**Short description**	**Problem formulation**		
		**Single-objective**	**Ref.**	**Multi-objective**
LPN3B	Linear pathway with three enzymatic reactions	Min. final time	[7]	Min. final time and intermediate consumption
LPDN4	Linear pathway with four enzymatic reactions	Min. final time plus enzyme consumption	[6]	Min. final time and enzyme consumption
GDB	Branched pathway with four enzymatic reactions	Min. final time	[15]	a) Min. final time and enzyme consumption
				b) Max. concentration of pathway products
S C	Simplified model of the central metabolism of *Saccharomyces cerevisiae*	Max. survival time	[3,4]	Max. survival time and min. enzyme cost

### Three-step linear pathway with mass action kinetics (LPN3B)

**Figure 1:**
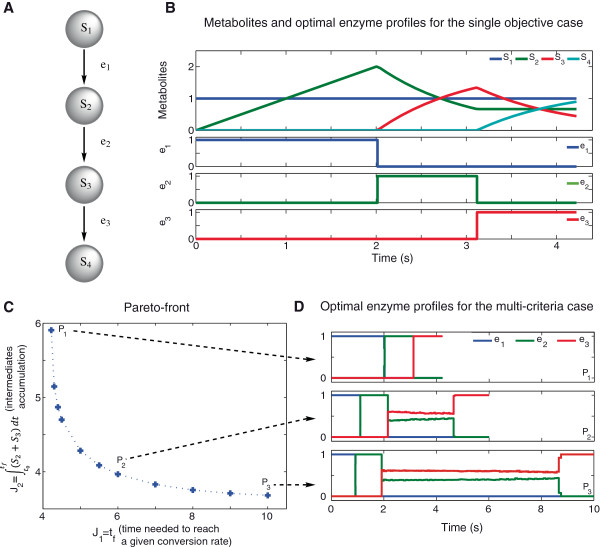
**Three-step linear pathway with mass action kinetics.A)** Schematic representation of the three-step linear pathway. The pathway converts the substrate (*S*_1_) into the product (*S*_4_) using three steps, each one catalyzed by a specific enzyme (*e*_*i*_). **B)** Metabolite dynamics and enzyme profiles that minimize the time needed to reach a certain amount of product with buffered concentration of substrate (LPN3B) as presented in Equation (10). **C)** Pareto front obtained for the minimization of time needed to reach a certain amount of product and the intermediates concentrations as shown in Equation (15). **D)** Enzyme profiles for the selected points of the Pareto front. Control variables have been approximated using *ρ*=120 steps with fixed duration. Metabolites and enzymes are expressed in concentration units and time in seconds (s).

This example was originally formulated by Bartl et al. [7] and solved by means of the maximum principle of Pontryagin. The pathway (Figure 1A) consists of three enzymatic reactions with mass action kinetics, each reaction catalyzed by a specific enzyme (*e*_*i*_). *S*_1_ corresponds to the substrate, *S*_2_ and *S*_3_ to the intermediate metabolites and *S*_4_ to the product. Metabolites and enzymes are expressed in concentration units and time in seconds.

In this work we considered the objective of the minimization of the time needed to reach a certain amount of product (Equation (10)), when the substrate remains unalterable (buffered substrate concentration) as represented in matrix *N*. This objective reflects a biological situation where there is no need to convert all the substrate into the product but a certain amount is essential.

The mathematical statement of the single-objective problem is as follows.

Find **e** over *t*∈[ *t*_*o*_,*t*_*f*_] to minimize:

(10) $$J=t_{f}$$

Subject to the system dynamics:

(11) $$\frac{dS}{\text{dt}}=N\upsilon$$

Where **N**:

$$N=\left[\begin{array}{lll} 0 & 0 & 0\\ 1 & -1 & 0\\ 0 & 1 & -1\\ 0 & 0 & 1 \end{array}\right]$$

And:

(12) $$\upsilon_{i}=k_{i}\cdot S_{i}\cdot e_{i}$$

With the following end-point constraint:

(13) $$S_{4}(t_{f})=P(t_{f})$$

and path constraint:

(14) $$\overset{3}{\underset{i=1}{\sum }}e_{i}\leq E_{T}$$

with: *E*_*T*_=1M, *k*_*i*_=1 *s*^−1^*i*=2,3,4, *S*_1_(*t*_0_)=1M, *S*_*i*_(*t*_0_)=0 for *i*=2,3,4 and *P*(*t*_*f*_)=0.9 M.

The above problem was solved with several methods, with the eSS scatter search solver as the best performer. Detailed results, including a discussion on multimodality, are given in Additional file 1: Tables S.6 and S.7.

Figure 1B presents the corresponding metabolite dynamics and optimal profiles of the enzymes for the single-objective optimization problem presented in Equation (10). The solution was obtained under the assumption of no substrate consumption (for example, in a constant culture medium). The first phase of the process is devoted to produce *S*_2_ as fast as possible, the enzyme is fully activated and since substrate is not consumed a high amount of *S*_2_ is produced and accumulated. Similarly the second and third phases correspond to a full activation of enzymes *e*_2_ and *e*_3_ respectively. The process time is *t*_*f*_=4.2*s*. It should be remarked that the optimal solution for this case was achieved by eSS in less than 30 *s* of computation in a standard PC. Sequential hybrids of SRES and DE achieved similar values but requiring longer computation times.

The optimal solution of the above single-objective problem (see Equation (10)) results in a significant accumulation of intermediates at the end of the process which may be harmful for the cell. Thus, we formulated a generalized problem so as to find the best compromise between the time required to achieve a certain amount of product and the intermediates accumulation. Mathematically the formulation of this multi-objective problem is:

Find **e** over *t*∈ [ *t*_*o*_,*t*_*f*_] to minimize:

(15) $$J=\;[\;t_{f},\int_{t_{0}}^{t_{f}}(S_{2}+S_{3})\text{dt}]$$

subject to the system dynamics presented in Equation (11), and with the constraints presented in Equations (13)–(14). The obtained Pareto front for the multi-objective problem (Equation (15)) is presented in Figure 1C. It can be observed that for process durations longer than 8 seconds the effect on the intermediate concentration is negligible, which means that no further improvements in the reduction of intermediates concentration can be achieved to satisfy the required amount of product.

To illustrate the differences in enzyme activation profiles for different trade-offs, Figure 1D presents the optimal profiles for three solutions from the Pareto front (*P*_1_ and *P*_3_ as extreme points, and *P*_2_ as a balanced trade-off between objectives). It can be noticed that the first enzyme is always fully activated, and its activation time is reduced when both objectives are considered (*P*_2_ and *P*_3_). It can also be noted that an intermediate zone is generated where both enzymes are activated. In this region *S*_2_ and *S*_3_ are produced and consumed in order to avoid their accumulation in the pathway. Additionally it is observed that in the final stage the third enzyme is fully activated to obtain the desired amount of product (*S*_4_).

### Four-step linear pathway with Michaelis-Menten kinetics (LPDN4)

**Figure 2:**
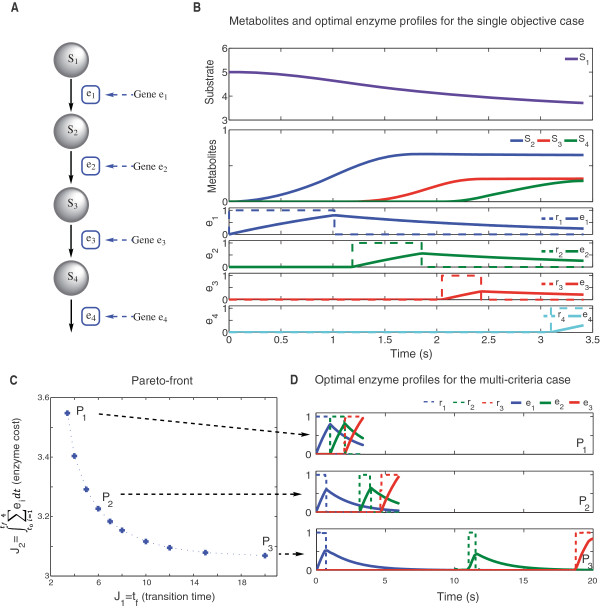
**Four-step linear pathway with Michaelis-Menten kinetics.A)** Schematic representation of a four-step linear pathway coupled with enzyme dynamics. It consists in four enzymatic reactions, where the substrate (*S*_1_) is converted into the product (*S*_4_). **B)** Metabolite dynamics, expression rates and enzyme activation profiles that minimize the time needed to reach a given steady state and the corresponding enzyme consumption, see Equation (16). **C)** Pareto front obtained by minimizing the time and the enzyme cost needed to reach a certain amount of product using Equation (23). **D)** Enzyme profiles for the points *P*_1_, *P*_2_ and *P*_3_ of the Pareto front using *ρ*=120 steps with fixed duration. Enzyme and metabolite concentrations are expressed in concentration units (mM) and time in seconds (s).

This example considers a four step linear pathway with Michaelis-Menten kinetics and was originally considered by Oyarzún et al. [6]. In this case, in order to obtain more realistic results, enzymes are assumed not to become activated instantaneously, but to follow first order kinetics. The pathway (Figure 2A) consists in four enzymatic reactions catalyzed by a specific enzyme (*e*_*i*_) where *S*_1_ corresponds to the substrate, *S*_2_−*S*_3_ to the intermediate metabolites and *S*_4_ to the product. The objective of the system is to minimize the enzyme consumption and the time needed to reach a given steady state.

This single-objective problem reads mathematically as follows:

Find **r** over *t*∈[ *t*_*o*_,*t*_*f*_] to minimize:

(16) $$J=\int_{t_{0}}^{t_{f}}(1+\overset{n=4}{\underset{i=1}{\sum }}e_{i})\text{dt}$$

Subject to the system dynamics:

(17) $$\frac{dS}{\text{dt}}=N\upsilon$$

where the enzyme dynamics is considered to be linear with the expression rate (**r**) and *λ*=0.5.

(18) $$\frac{de}{\text{dt}}=r-\lambda \cdot e$$

with:

$$N=\left[\begin{array}{llll} -1 & \;\;\;\;0 & \;\;\;\;0 & \;\;\;\;0\\ \;\;\;\;1 & -1 & \;\;\;\;0 & \;\;\;\;0\\ \;\;\;\;0 & \;\;\;\;1 & -1 & \;\;\;\;0\\ \;\;\;\;0 & \;\;\;\;0 & \;\;\;\;1 & -1 \end{array}\right]$$

and:

(19) $$\upsilon_{i}=\frac{k_{\text{cati}}\cdot S_{i}}{K_{M}+S_{i}}\cdot e_{i}$$

with the following end point constraints, which describe the given steady state.

(20) $$e(t_{f})=e_{\text{SS}}$$

In addition the following constraints are imposed to limit the amount of enzymes and their rates:

(21) $$0\leq r_{i}\leq 1\;\mathrm{mM.}s^{-1}$$

(22) $$0\leq e_{i}\leq E_{t}$$

With: *E*_*T*_=1 *mM*, *k*_*c* *a* *t*1_=1 *s*^−1^, *k*_*c* *a* *t*2_=2 *s*^−1^, *k*_*c* *a* *t*3_=4 *s*^−1^, *k*_*c* *a* *t*4_=3 *s*^−1^, *K*_*M*_=1 *s*^−1^, *V*=0.2 *mM*/*s*, *S*_1_(*t*_0_)=5*mM*, *S*_*i*_(*t*_0_)=0 for *i*=2,3,4, **e**_*SS*_= [0.24 0.26 0.21 0.29] *mM* for *i*=1,2,3,4.

Note that in this case the expression rates are computed via dynamic optimization and the optimal enzyme activation profiles are obtained from the Equation (18).

The optimal value *J*^∗^ = 6.154 was obtained using eSS. Note that this result is in good agreement with the one previously reported (*J*^∗^ = 6.298) [35]. Figure 2B presents the optimal metabolite and enzyme dynamics for the single-objective case (Equation (16)). As expected, the incorporation of the enzyme dynamics slows down the entire process. The optimal profiles for the expression rates follow a switching pattern that matches the pathway topology, leading to enzyme profiles that follow a sequential activation in agreement with previous results. The enzyme profiles show that for the synthesis of one enzyme the degradation of the previous enzyme is needed. The constraints imposed in the final amount of metabolites require a high accumulation of metabolites in the system and this could be lethal for the system [36].

It is interesting to note that in two previous works [6,35] a combined objective function including the transition time and the enzyme cost was proposed, obtaining a single trade-off solution. In other words, they obtained one of all the possible Pareto solutions. Here we go a step a further and formulate and solve the multi-objective optimization problem in order to obtain the full Pareto front. Mathematically the formulation of the multi-objective problem is:

Find **e** over *t*∈[ *t*_*o*_,*t*_*f*_] to minimize:

(23) $$J=[\;t_{f},\int_{t_{0}}^{t_{f}}(\overset{n=4}{\underset{i=1}{\sum }}e_{i})\cdot \text{dt}]$$

Subject to Equations (17)–(18) and with the constraints presented in Equations (21)–(22).

Due to the final time constraints used to define the steady state the system keeps several enzymes activated during the process, which implies an extra effort for the system as discussed in literature [7]. In this case end point constraints (Equation (20)) are replaced by a constraint on the final amount of product:

(24) $$S_{4}(t_{f})=0.7$$

The Pareto front for the multi-objective case (Equation (23)) is presented in Figure 2C. It can be noticed that for large times (around 15 seconds) the effect on the reduction of enzyme concentration is negligible, which means that the enzyme consumption can not be further reduced to achieve the desired amount of product. In Figure 2D optimal profiles for expression rates and enzyme dynamics are shown in order to check how enzyme activation is affected by the different trade-off. *P*_1_ corresponds to the minimization of the time and *P*_2_ and *P*_3_ to different trade-off between objectives.

We also notice that the optimal profiles for the expression rates follow a switching pattern that matches with the pathway topology, i.e. is reflected in a sequential activation of the enzymes. Before activating one enzyme, the degradation process of the previous one has started (i.e. the cell has only a certain amount of protein available). This situation is more relevant when the enzyme consumption is reduced. Note that when the process time increases the activation time of the enzymes is slightly reduced (this decrease is more relevant for short process times).

### Glycolysis inspired network (GBD)

**Figure 3:**
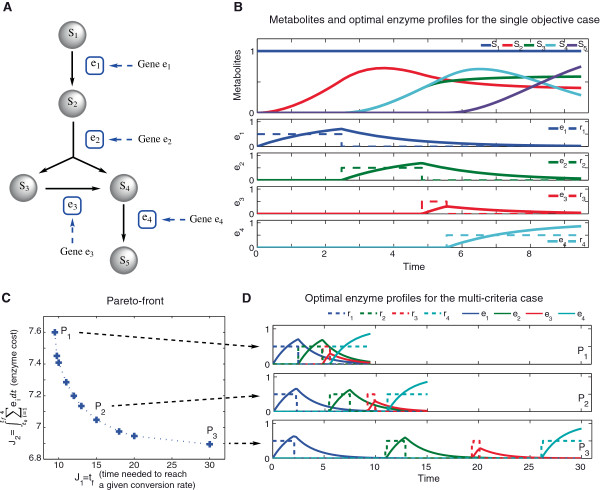
**Glycolysis inspired network.A)** Schematic representation: The pathway consists in four enzymatic reactions with one branch where *S*_1_ corresponds to the substrate, *S*_2_−*S*_4_ to the intermediate metabolites and *S*_5_ to the product. **B)** Optimal profiles for the Glycolysis inspired pathway with inexhaustible substrate (single-objective case, Equation (25)), obtained with eSS. **C)** Resulting Pareto front for the minimization of the time and the enzyme cost needed to achieved a certain amount of product in a branched pathway coupled with enzyme dynamics using Equation (31). **D)** Optimal control profiles for enzyme activation for different points of the Pareto front, were approximated using *ρ*=120 steps with fixed duration. Enzyme and metabolite concentrations are expressed in concentration units (mM) and time in seconds (s).

The original problem was considered by Bartl et al. [15]. Results achieved for that formulation are discussed in Additional file 1. Here we propose a more realistic formulation that incorporates the enzyme dynamics. The enzyme dynamics (Equation (27)) are considered to be linear with the expression rate (*r*_*i*_). The pathway (Figure 3A) consists of four enzymatic reactions with one branch where *S*_1_ corresponds to the substrate, *S*_2_−*S*_4_ to the intermediate metabolites and *S*_5_ to the product. The hypothesis in this problem is that the pathway activation minimizes the time needed to transform the substrate (*S*_1_) into a fixed amount of product (*S*_5_). Note that the substrate *S*_1_ is not consumed during the process.

The activation profiles for the single-objective case may be acquired by computing **r** over *t*∈ [ *t*_*o*_,*t*_*f*_] to minimize the cost function.

(25) $$J=t_{f}$$

Subject to the system dynamics:

(26) $$\begin{array}{lcr} & & \frac{dS}{\text{dt}}=N\upsilon \end{array}$$

(27) $$\begin{array}{lcr} & & \frac{de}{\text{dt}}=r-\lambda \cdot e \end{array}$$

Where:

(28) $$\upsilon_{i}=\frac{k_{\text{cati}}\cdot S_{i}}{K_{M}+S_{i}}\cdot e_{i}$$

With:

$$N=\left[\begin{array}{llll} 0 & \;\;\;\;0 & \;\;\;\;0 & \;\;\;\;0\\ 1 & -1 & \;\;\;\;0 & \;\;\;\;0\\ 0 & \;\;\;\;1 & -1 & \;\;\;\;0\\ 0 & \;\;\;\;1 & \;\;\;\;1 & -1\\ 0 & \;\;\;\;0 & \;\;\;\;0 & \;\;\;\;1 \end{array}\right]$$

and the following end point constraints:

(29) $$S_{5}(t_{f})=P(t_{f})$$

and the following path constraint:

(30) $$\overset{4}{\underset{i=1}{\sum }}e_{i}\leq E_{T}$$

With *E*_*T*_=1*mM*, *P*(*t*_*f*_)=0.75*mM*, *k*_*cati*_=1 *s*^−1^, *K*_*M*_=1 *s*^−1^, *S*_1_(*t*_0_)=1*mM*, *S*_*i*_(*t*_0_)=*e*_*i*_(*t*_0_)=0 for *i*=2,3,4,5, *λ*=0.5 *s*^−1^.

The optimal value (*J*^∗^=9.5) was obtained using scatter search (eSS) in less than 50 *s* of CPU time. Detailed optimization results are presented in Additional file 1 (Table S.13). The corresponding optimal enzyme activation profiles for the single-objective case (Equation (25)) are shown in Figure 3B. Again the optimal profiles for the expression rate follow a switching pattern that matches with the pathway topology leading to enzyme profiles that follow a sequential activation profile.

The enzyme profiles show that for the synthesis of one enzyme the degradation of the previous is needed, meeting the constraint on the total amount of enzyme (Equation (30)). As in previous cases, there is a high accumulation of metabolites, which could be harmful for the cell. Note that the problem formulation could be modified so to predict the scenario with no accumulation of intermediates. In addition, these calculations were preformed for a fixed *λ* value, but of course the problem can be easily solved to consider other cases where genes might have different expression rates and proteins might have different degradation rates.

We now consider a multi-objective formulation, extending the objective function and keeping unchanged the rest of the problem. Mathematically, the multi-objective problem is formulated as follows:

Find **r** over *t*∈ [ *t*_*o*_,*t*_*f*_] to minimize:

(31) $$J=[\;t_{f},\int_{t_{0}}^{t_{f}}(\overset{4}{\underset{i=1}{\sum }}e_{i})\cdot \text{dt}]$$

The Pareto front is presented in Figure 3C for the objectives in Equation (31). For long process times, no significant improvement in the enzyme cost is achieved. The optimal enzyme profiles corresponding to 3 points in the Pareto front are depicted in Figure 3D. *P*_1_ corresponds to the single-objective case (time minimization), and *P*_2_ and *P*_3_ represent different trade-offs between process time and enzyme cost minimization. The optimal profiles for the expression rate follow a switching pattern that matches with the pathway topology. In *P*_1_ we observed that for the synthesis of one enzyme the degradation of the previous is needed. In fact, from *P*_2_ and *P*_3_ it can be noted that such situation gains relevance as we move in the Pareto front.

**Figure 4:**
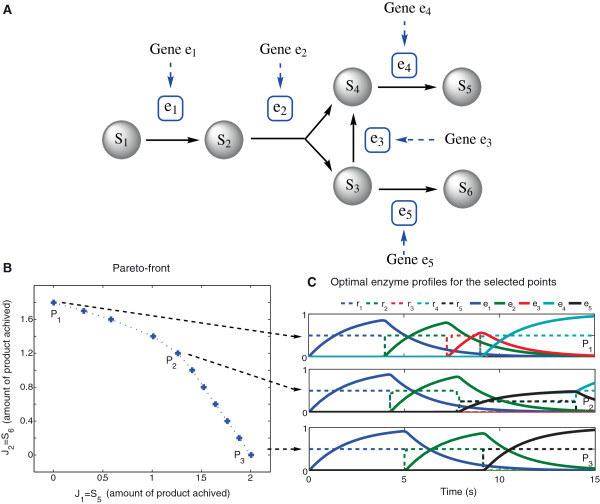
**Glycolysis inspired network with two outputs.A)** Schematic representation of the branched pathway with two outputs (*S*_5_ and *S*_6_). **B)** Pareto front obtained for the maximization of both products of a branched pathway (*S*_5_ and *S*_6_) using Equation (32). **C)** Optimal enzyme profiles for the maximization of the two different outputs of a pathway (*P*_1_, *P*_2_ and *P*_3_). Control variables have been approximated using *ρ*=120 steps with fixed duration. Enzyme and metabolite concentrations are expressed in concentration units (mM) and time in seconds (s).

One interesting and common situation in branched pathways is that the system could have two different outputs (p.e. produce several amino acids in sensible ratios for protein synthesis), which in practice means that the cell resources are distributed accordingly. The introduction of a second output in the single-output pathway was considered to study the behavior of the system if several products are generated. This new pathway is presented in Figure 4A.

The new multi-objective problem mathematically reads as: find activation profiles by computing **r** over *t*∈[ *t*_*o*_,*t*_*f*_] to maximize:

(32) $$J=\;[\;S_{5},S_{6}]$$

Subject to the system dynamics presented in Equations (26)–(27), with:

$$N=\left[\begin{array}{llll} 0 & \;\;\;\;0 & \;\;\;\;0 & \;\;\;\;0\\ 1 & -1 & \;\;\;\;0 & \;\;\;\;0\\ 0 & \;\;\;\;1 & -1 & -1\\ 0 & \;\;\;\;1 & \;\;\;\;1 & -1\\ 0 & \;\;\;\;0 & \;\;\;\;0 & \;\;\;\;1\\ 0 & \;\;\;\;0 & \;\;\;\;0 & \;\;\;\;1 \end{array}\right]$$

And with the path constraint presented in (30). In this case the final time was considered constant (*t*_*f*_=15 s). The Pareto front for the problem formulated in Equation (32) is presented in Figure 4B, where point *P*_1_ and *P*_3_ stand for single-objective situations were only one product is produced, and *P*_2_ is a balanced trade-off. In Figure 4C the optimal profiles of the selected Pareto points (*P*_1_, *P*_2_ and *P*_3_) are depicted. In all cases we can see a sequential enzymatic activation. In *P*_1_ four enzymes are activated to convert the substrate (*S*_1_) in the product *S*_5_. In *P*_3_ only 3 enzymes are activated to obtain the product *S*_6_. Regarding *P*_2_, it can be noted that *e*_1_ and *e*_2_ are sequentially activated during the early process. Later on, in the intermediate stage, *e*_4_ and *e*_5_ are simultaneously activated. And finally, at the end of the process, only *e*_4_ is active.

### Central metabolism of ***Saccharomyces cerevisiae*** during diauxic shift (SC)

The diauxic shift is characterized by decreased growth rate and by switching metabolism from glycolysis to aerobic utilization of ethanol under conditions of glucose depletion. This allows the maintenance of the cellular redox potential, NADH/NAD and ATP levels, enabling the cell to survive over longer periods without nutrients, in a so called quiescent state. The idea is to explain this particular behavior by computing the enzymatic activation profile that maximizes the survival time.

**Figure 5:**
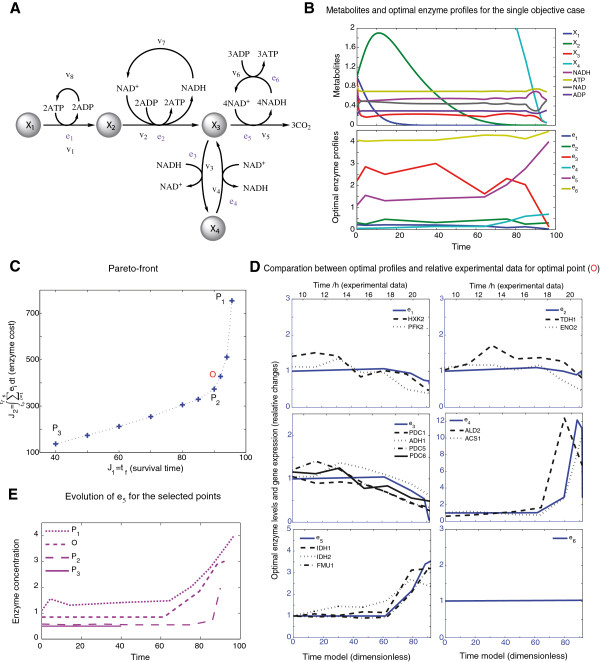
**Central metabolism of *Saccharomyces cerevisiae. *during diauxic shift.A)** Scheme of pathway of the central metabolism of *Saccharomyces cerevisiae*. **B)** Metabolite dynamics and enzyme activation profiles corresponding to the maximization of the survival time of *S. cerevisiae* under diauxic shift (see Equation (33)). Metabolites and enzymes are expressed in arbitrary units. **C)** Pareto front for the maximization of the survival time and the minimization of the enzyme consumption of *S. cerevisiae* under diauxic shift (Equation (40)). Optimal profiles have been approximated to 8 linear elements with variable length. **D)** Comparison between experimental data and model predictions for the SC example for point O. **E)** Evolution of *e*_5_ for the selected points.

This case was considered by Klipp et al. [3,4] using orthogonal collocation in combination with a genetic algorithm. The pathway (Figure 5A) consists in a simplified model of the central metabolism of *Saccharomyces cerevisiae*. The system complies six reactions: upper glycolysis, lower glycolysis, ethanol formation, ethanol consumption TCA cycle and respiratory chain. The model has arbitrary units.

Mathematically the single-objective problem is formulated as follows:

Find **e** over *t*∈ [ *t*_*o*_,*t*_*f*_] to maximize:

(33) $$J=t_{f}$$

Subject to the system dynamics:

(34) $$\frac{dS}{\text{dt}}=N\upsilon$$

Where **S** and **N** correspond to:

(35) $$S={\left[X_{1},X_{2},X_{3},X_{4},\text{NADH},\text{ATP},\text{NAD},\text{ADP}\right]}^{T}$$

$$N=\left[\begin{array}{llllllll} -1 & 0 & 0 & 0 & 0 & 0 & 0 & 0\\ 2 & -1 & 0 & 0 & 0 & 0 & 0 & 0\\ 0 & 1 & -1 & 1 & -1 & 0 & 0 & 0\\ 0 & 0 & 1 & -1 & 0 & 0 & 0 & 0\\ 0 & 1 & -1 & 1 & -4 & -1 & -1 & 0\\ -1 & -2 & 0 & 0 & 0 & 3 & 0 & -1\\ 0 & -1 & 1 & -1 & 4 & 1 & 1 & 0\\ 1 & 2 & 0 & 0 & 0 & -3 & 0 & 1 \end{array}\right]$$

And:

(36) $$\begin{array}{lcll} \upsilon_{1} & = & e_{1}\cdot X_{1}\cdot \text{ATP} & \\ \upsilon_{2} & = & e_{2}\cdot X_{2}\cdot \text{NAD}\cdot \text{ADP} & \\ \upsilon_{3} & = & e_{3}\cdot X_{3}\cdot \text{NADH} & \\ \upsilon_{4} & = & e_{4}\cdot X_{4}\cdot \text{NAD} & \\ \upsilon_{5} & = & e_{5}\cdot X_{3}\cdot \text{NAD} & \\ \upsilon_{6} & = & e_{6}\cdot \text{NADH}\cdot \text{ADP} & \\ \upsilon_{7} & = & k_{7}\cdot \text{ATP} & \\ \upsilon_{8} & = & k_{8}\cdot \text{NAD} \end{array}$$

With a constraint in the total amount of enzyme:

(37) $$\overset{6}{\underset{i=1}{\sum }}e_{i}\leq E_{T}$$

And:

(38) $$\begin{array}{lc} & \text{NADH}\geq {\text{NADH}}_{c} \end{array}$$

(39) $$\begin{array}{lc} & \text{ATP}\geq {\text{ATP}}_{c} \end{array}$$

With: *X*_1_(*t*_0_)=1, *X*_2_(*t*_0_)=1, *X*_3_(*t*_0_)=1, *X*_4_(*t*_0_)=10, *NADH*(*t*_0_)=0.7, *NAD*(*t*_0_)=0.3, *ATP*(*t*_0_)=0.8, *ADP*(*t*_0_)=0.2, *NADH*_*c*_=0.5, *ATP*_*c*_=0.7, *k*_7_=3 and *k*_8_=0.1.

It should be noted that constraints (38)–(39) indicate that the cells will survive provided the concentrations of NADH and ATP are above some given critical values (NADH _*c*_ and ATP _*c*_ respectively).

In order to compare our results with the ones reported in the work by Klipp et al. [4] and the available experimental data, two types of enzyme CVP approximations were considered: i) a step-wise approximation with 120 constant elements for each enzyme (i.e. 721 decision variables) and ii) a piecewise linear approximation with 8 variable length linear elements (i.e. 55 decision variables).

The optimal value (J* = 95.8) was again obtained using eSS scatter search. Note that the optimal solution is highly sensitive to the switching times (details are given in Additional file 1). The optimal profiles and the metabolite dynamics for the single-objective problem (Equation (33)) are depicted in Figure 5B, showing a higher activity of the lower part of the glycolysis (*e*_2_) and in the ethanol formation (*e*_3_), as expected. During the last part of the process the activity of (*e*_4_) is increased allowing the formation of NADH. Also there is an increase in the tricarboxylic acid cycle (*e*_5_) and in the respiratory chain (*e*_6_) which compensate the decline in supply of NADH and ATP.

In the above single-objective formulation (Equation (33)), we can interpret that the system tries to survive as long as possible without any limitation on the investment of resources used. In the next case, we try to find the best compromise between the survival of the system and the resources used to do that. The aim in this case is to maximize the survival time with a low investment on the enzyme cost. We formulate the optimization problem as a multi-objective problem (with objectives time and the enzyme cost). Mathematically the multi-objective problem is formulated as follows:

Find **e** over *t*∈ [ *t*_*o*_,*t*_*f*_] to maximize:

(40) $$J=[\;t_{f},-\int_{t_{0}}^{t_{f}}(\overset{6}{\underset{i=1}{\sum }}e_{i})\text{dt}]$$

The set of solutions for the maximization problem (Equation (40)) is presented in Figure 5C. *P*_1_ corresponds to the single-objective situation above, while *P*_2_, *P*_3_ and *O* are different trade-offs among the objectives. There, we can observe that the survival time of the system is not significantly reduced by a decrease on the enzyme consumption until the reduction on the enzyme consumption is over 60%. For low enzyme consumptions (under 60%), the survival time is clearly affected and diminishes rapidly.

The reduction on enzyme consumption is reflected as a reduction on the initial value of enzymes and their dynamics during the process. In Figure 5E optimal profiles for *e*_5_ obtained for four different points of the Pareto front are compared. Besides the reduction on the initial concentration of the enzyme, it can be observed that in *P*_1_ and *O* there is a sharp increase on the activity of the enzyme towards then end of the process. This dynamic is significantly reduced in *P*_2_, and disappears in *P*_3_, where the enzyme has a constant activation value. Complete optimal profiles are presented in Additional file 1.

We computed, for all the Pareto points, the distance between experimental data (taken from [4]) and model predictions, finding that point “O” in the Pareto corresponds to the best fit. Figure 5D presents the comparison between experimental data and model predictions for this optimal trade-off solution. The numerical profiles are in good agreement with the general tendency of the experimental data. *e*_1_ and *e*_3_ slowly decrease the activity whereas *e*_4_ and *e*_5_ slowly increase their activity arriving to high activation values. *e*_2_ keeps an almost constant activation value along the time horizon.

## Conclusions

This work proposes the use of multi-objective optimization, combined with an advanced dynamic optimization method, as a systematic way to predict or explain genetic metabolic regulation. The presented methodology can handle very general formulations, including several objectives, arbitrary network topologies and non-linear kinetics, large numbers of control variables (enzymes or activation rates), and general path and point constraints on controls and states (metabolites concentrations).

The methodology is based on the transformation of the original multi-objective dynamic optimization problem into a set of non-linear programming (NLP) problems by means of the control vector parametrization. The NLP problems can be solved using the combination of an initial value problem solver and an efficient global optimizer. The need of global optimization arises from the non-convexity of these problems.

To illustrate this methodology, two sets of problems were considered. A first set of single-objective examples were taken from the literature. The obtained results are comparable or better than those previously published and were obtained with a minimum computational cost, between a few seconds and a few minutes in a standard PC. Our results show clearly that a hybrid metaheuristic based on scatter search was the most robust and efficient solver when dealing with this class of problems.

A second set of multi-objective formulations were considered, for the first time, to achieve more biologically meaningful results. The Pareto front which represents the different trade-offs between objectives was obtained by transforming the multi-criteria problem into a set of single-objective problems, each of them solved by means of the previously mentioned methodology. Interestingly, the optimal activation profiles computed exhibited the *“just-in-time”* behavior in most cases and for both single and multi-criteria formulations. By definition, all the solutions in a Pareto front are equally optimal. However, if one introduces an additional requirement or constraint (e.g. fit to experimental data), it is then possible to select a single solution from the front which meets such additional criteria (as illustrated with the *S. cerevisiae* case study).

The methodology presented here opens up new possibilities, especially with respect to the handling of medium and large scale complex networks. It will also enable further research, including experimental validation, in order to asses biological optimality in terms of Pareto-optimal trade-offs.

## Competing interests

The authors declare that they have no competing interests.

## Authors’ contributions

GMHL performed the computations. EBC implemented the dynamic optimization code. EBC and JRB contributed to the conception and design of the work. EBC, JRB and EK supervised the project and helped to draft the manuscript. All authors read and approved the final manuscript.

## Supplementary Material

Additional file 1 **Further details on the use of global dynamic optimization to predict the activation in metabolic pathways.** The Additional file 1 presents a more detailed description of the numerical approaches used in this work as well as a comparative study of the results achieved. Click here for file
