# Supplementary material for: Global dynamic optimization approach to predict activation in metabolic pathways
Source: BMC Syst Biol. 2014 Jan 6;8:1. doi: 10.1186/1752-0509-8-1 (PMC3892042; doi:10.1186/1752-0509-8-1)
Supplement: Additional file 1 — Further details on the use of global dynamic optimization to predict the activation in metabolic pathways. The Additional file 1 presents a more detailed description of the numerical approaches used in this work as well as a comparative study of the results achieved. [file 1752-0509-8-1-S1.pdf]

Additional file 1

Further details on the use of global dynamic optimization to predict the activation in metabolic pathways

G. M. de Hijas-Liste[1], *E. Klipp*[2], *E. Balsa-Canto*[1] *and J. R. Banga*[1]

[1] Bioprocess Engineering Group, Spanish National Research Council, IIM-CSIC, C/Eduardo Cabello 6, 36208 Vigo (Spain)

[2] Theoretical Biophysics, Humboldt-Universität zu Berlin, Invalidenstr. 42, 10115 Berlin (Germany)

This additional file presents the results achieved when a considering a set of single-objective problems taken from literature [1–5]. First, we analyze the multimodal nature of the NLPs by means of a multistart approach. After concluding on the multimodal nature of the cases comparation of global optimization methods is performed to conclude that eSS offers the best compromise between efficiency and robustest. First section discusses details on the selected CVP approach. Second section presents the NLP methods both local and global considered. Following sections present results obtained for different case studies.

Details on the implementation of the control vector parameterization approach

As explained in the main text the control vector parameterization (CVP) method was selected in this work as a means to approximate control variables ($\mathbf{e}$ or $\mathbf{r}$). In many of the cases considered the activation profiles are known to be bang-bang (i.e. from full activation to deactivation or the other way around) or step-wise. It is well known that for those type of profiles the computation of the switching points, i.e. when the enzyme change between two different levels, is critical. In this sense, two possibilities exist, either to use a large control discretization level (PWC-f), where decision variables correspond to the control levels, or to introduce the switching points as decision variables (PWC-v). Remark that the correct approximation of the switching points may require an excessively large discretization making the associated non-linear programming problem (NLP) too computationally demanding. To illustrate this point we present here the evolution of the results obtained for the single-objective examples GUB (Table S.1) and LPN3B (Table S.2) using different control approximations. As an illustrative example in Figure S.1 optimal profiles for enzyme activation obtained with PWC-f and ρ =80 are shown.

In the single-objective formulation of SC example, related to the central metabolism of *Saccharomyces cerevisiae* during diauxic shift, available experimental data reveal a different type of enzyme activation profile. In this scenario, two possibilities exist, either to use a large discretization step-wise approximation of the enzymes profiles or to use a piece-wise linear interpolation with varying length elements (PWL-v). It should be noted that PWL-v interpolations allow to significantly reduce the number of decision variables and therefore the computational effort.

[1]In general, objective function values are given using the decimal figures provided by the optimization tolerance (10^{-5})
[2]Computer: Intel(R) Core(TM)2 Quad CPU Q9550 2.83GHz, 3.25 GB RAM.

CVP	Best (min)[1]	Worst	Mean	CPU time (s)[2]
PWC-f 10	7.88883	7.88903	7.88896	83.4
PWC-f 25	7.86248	7.86518	7.86303	202
PWC-f 50	7.85896	7.85999	7.85937	405
PWC-f 100	7.85877	7.85937	7.85896	880
PWC-v 4	7.85778	7.85778	7.85778	40.7

Table S.1: **Effect of the CVP selection in the optimal solution, finer PWC-f approximations as compared to coarse PWC-v in the GUB single-objective example.** The solution improves as soon as the discretization level increases in the PWC-f approach whereas the best solution is achieved when the switching points are incorporated as decision variables in the PWC-v. It should be noted that the computational effort is substantially reduced when using PWC-v.

CVP	Best (min)[1]	Worst	Mean	CPU time (s)[2]
PWC-f 10	4.24163	4.24363	4.2419	122.1
PWC-f 20	4.22632	4.23633	4.22890	254
PWC-f 40	4.22286	4.23288	4.22491	355
PWC-f 80	4.22210	4.22216	4.22212	760
PWC-v 1	7.32493	7.32493	7.32493	16.5
PWC-v 2	5.00245	5.00245	5.00245	21.2
PWC-v 3	4.22210	4.22214	4.22212	26.5
PWC-v 5	4.22210	4.22215	4.22212	88.7

Table S.2: **Effect of the CVP selection in the optimal solution, finer PWC-f approximations as compared to coarse PWC-v in the LPN3B single-objective example.** The optimal value obtained improves when the discretization level increases in the PWC-f approach. Note that for the PWC-v, the solution is optimal when the number of variable functions is approximated to the number of step of the pathway, three in this case.

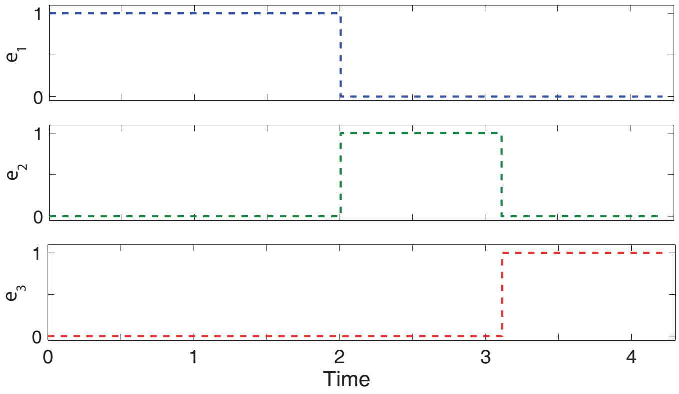

Figure S.1: **Enzyme profiles corresponding to the single-objective problem LPN3B obtained with PWC-f and ρ =80 where the expected three switches were achieved**. Metabolites and enzymes are expressed in concentration units and time in seconds (s).

In the control vector parameterization approach an initial value problem solver is required so as to handle the dynamic constraints. Here RADAU5 [6], an implicit Ruge-Kutta method, was used. For all examples

the integration tolerances (absolute and relative) were 10^{-7}.

Table S.3 presents a summary of the formulation of the problems considered with the CVP type select for their solution.

Problem label	Ref.	Objective	Controls	NLP		
				CVP	Decision variables	Constraints
LPN3tt	[4]	$min(\int_{t_0}^{t_f} \sum_{i=1}^{n=3} S_i dt)$	e_i	3 PWC-v	12	4
LPN3UB	[4]	$min(t_f)$	e_i	3 PWC-v	12	4
LPN3B	[4]	$min(t_f)$	e_i	3 PWC-v	12	4
LPN5	[2]	$min(\int_{t_0}^{t_f} (1 - S_6)dt)$	e_i	5 PWC-v	30	6
LPN4	[3]	$min(\int_{t_0}^{t_f} (1 + \sum_{i=1}^{n=4} \mathbf{e}_i)dt)$	e_i	4 PWC-v	20	8
LPDN4	[3]	$min(\int_{t_0}^{t_f} (1 + \sum_{i=1}^{n=4} \mathbf{e}_i)dt)$	r_i	7 PWC-v	40	12
GUB	[5]	$min(t_f)$	e_i	4 PWC-v	20	5
GB	[5]	$min(t_f)$	e_i	4 PWC-v	20	5
GDUB	[5]	$min(t_f)$	r_i	4 PWC-v	20	5
GDB	[5]	$min(t_f)$	r_i	4 PWC-v	20	5
SC	[1]	$max(t_f)$	e_i	8 PWL-v	55	10

Table S.3: **Summary of formulation together with the CVP scheme used for each single-objective example.** Problem label corresponds to the short name given to each of the examples; Ref., regards the original source of the problem; Objective, corresponds to the performance index considered in each problem; Controls, correspond to the control variables enzymes (**e**) or rates (**r**) ; CVP, regards of the type of approximation used for the control variables, nPWC-v (n piece-wise constant elements of variable length) or nPWL-v (n piece-wise linear elements of variable length); decision variables and constraints, correspond to the total number of decision variables and constraints in the equivalent NLP.

Non-linear programming methods

As described in the main text we have considered the use of several local deterministic and global stochastic methods including hybrid methods. Methods were selected attending to their previously published records.

Local deterministic methods

- **fmincon** as implemented in MATLAB, uses a sequential quadratic programming (SQP) method using a BFGS approximation of the Hessian (see, for example, [7])
- **FSQP** [8] is an implementation of two algorithms based on SQP, modified so as to generate feasible iterates.
- **SOLNP** [9] is implemented in MATLAB and is based on a SQP approach which makes use of the BFGS update of the Hessian.
- **ACADO Toolkit** [10] which makes use of the multiple shooting approach in combination with several SQP methods.

multi-start of local methods. Often regarded as the simplest global approach, it is based on launching a local solver from a sufficiently large number of randomly selected initial guesses. The optimal solutions found may be plotted against the frequency with which they were achieved. If a unique solution is always obtained the problem is assumed to be convex, if a distribution of solutions appears the problem is considered to be multimodal.

Global stochastic methods

- **Stochastic Ranking Evolutionary Search, SRES** [11], the method is based on a (μ, λ) evolution strategy and introduces a novel approach to balance objective and penalty functions stochastically to handle constraints.

- **Differential evolution, DE** [12], is a population based approach which makes use of different strategies to select members of the population and generates new iterates by adding the weighted difference between two selected population vectors to a third selected vector.

Hybrid methods

- **Sequential hybrids** [13], in this case any combination of SRES or DE with the deterministic local methods mentioned above is possible. The idea is to use the global solver for a fixed number of iterations and from that solution call a local solver.

- **Enhanced scatter search, eSS** [14], is a population based method that shares some concepts of the scatter search metaheuristic [15] but includes fundamental modifications which improve the balance between intensification (i.e., efficiency) and diversification (i.e., robustness) of the search with a lower number of tuning-parameters. The method offers the possibility to call local deterministic methods from automatically selected initial points.

It should be noted that most optimization solvers performance depends on a number of method dependent tunning parameters. Table S.4 summarizes the values selected to solve all case studies considered in this work.

DE	SRES
NP=10$\times$*nvar*	λ=10$\times$*nvar*
strategy=3	μ=λ/7
VF=0.85	pf=0.45
CR=1	varphi=1

Table S.4: **Method specific parameters for DE and SRES.** NP and λ correspond to the population size; DE allows to select within 10 different strategies, the strategy 3 corresponds to a rand-to-best selection of population members to generate new iterates, VF has to do with the step size and CR with the crossover probability; default values have been selected for μ (number of parents), pf (pressure of fitness) and varphi (expected rate of convergence) in SRES.

In addition some method related parameters will be problem dependent. Table S.5 presents a summary of specific solver parameters used for each of the examples considered.

	LPN3	LPN5	LPN4	LPDN4	G	GD	SC
max iter (global)	1500	3500	1500	2500	2000	2500	4500
max iter (hybrid)	500	500	600	600	500	600	600
max feval (eSS)	40000	50000	80000	100000	60000	60000	60000
max CPU time (eSS)	$2\times nvar$	$3\times nvar$	$5\times nvar$	$7\times nvar$	$3\times nvar$	$3\times nvar$	$4\times nvar$

Table S.5: **Specific solver parameters used for each of the examples considered.** *max iter* corresponds to the maximum number of iterations for the global methods DE and SRES when used as global or within a sequential hybrid, note that this will correspond to a maximum of $maxiter \times 10 \times nvar$ cost function evaluations; *max feval* and *max CPU time* correspond to two stopping criteria incorporated in eSS, the method will stop when one of those criteria is verified.

Three-step linear pathway with mass action kinetics (LPN3B)

In the original work [4] the problem was additionally solved for a situation where the substrate is consumed during the reaction process ($\mathbf{N}_{UB}$), for this matrix they considered two different objective functions: minimization the transition time (LPN3tt) and minimization of the time needed to achieve a certain amount of product (LPN3UB). Problem formulations and solutions to these single-objective cases are presented here. Special attention will be paid to the multimodal nature of the problems. To that propose a multistart approach will be employed to study the all single-objective cases. Also in this section the optimization problems were solved with several nonlinear programing solvers.

Problem formulation and results for sub-case study of the minimization of the transition time (LPN3tt)

The objective in this case is the minimization of the transition time, as shown in Eqn. (1). The transition time is regarded as the time required to achieve a certain conversion of the substrate into the product (90% in this case) by means of the intermediates. The mathematical definition was drawn from the previous works [4, 16]. The mathematical statement is as follows.

Find $\mathbf{e}$ over $t \in [t_o, t_f]$ to minimize:

$$\tau = \int_{t_0}^{t_f} (S_1 + S_2 + S_3)dt \tag{1}$$

$$\tag{2}$$

Subject to the system dynamics:

$$\frac{d\mathbf{S}}{dt} = \mathbf{N}_{UB}\boldsymbol{\upsilon} \tag{3}$$

Where $\mathbf{N}_{UB}$:

$$\mathbf{N}_{UB} = \begin{bmatrix} -1 & 0 & 0 \\ 1 & -1 & 0 \\ 0 & 1 & -1 \\ 0 & 0 & 1 \end{bmatrix}$$

And:

$$\upsilon_i = k_i \cdot S_i \cdot e_i \tag{4}$$

With the following end-point constraint:

$$S_4(t_f) = P(t_f) \quad (5)$$

and path constraint:

$$\sum_{i=1}^{3} e_i \leq E_T \quad (6)$$

with: $E_T = 1$ M, $k_i = 1\ s^{-1}$ $i = 2, 3, 4$, $S_1(t_0) = 1$ M, $S_i(t_0) = 0$ for $i = 2, 3, 4$ and $P(t_f) = 0.9$ M.

The solution is approximated by 3 variable length steps with a constant value for each enzyme in each step. Thus the total number of decision variables is 12 (3 enzymes concentration times ·3 steps+3 switching times). The problem was initially solved with a multi-start of local solvers (Table S.6). And later, the obtained constrained NLP was solved with several nonlinear programing solvers. Results are reported in Table S.7.

The best solution for the case LPN3tt, $\tau = 6.6\ s$ (in general optimal time values were rounded to the nearest tenth), was achieved by eSS in half a minute whereas sequential hybrid methods took between 50 s and one minute of CPU time. Note that the best previous solution, $\tau = 7.2\ s$ was reported by Bartl et al. [4].

Figure S.2 presents the corresponding metabolite dynamics and optimal profiles of the enzymes. Results indicate that in the first phase of the process, the first enzyme (e_1) is activated, reaching the maximum allowed value, and all the resources of the pathway are devoted to transform the substrate (S_1) into the first intermediate (S_2) as efficiently as possible. Equivalently, the objective of the second phase, when enzyme two is fully activated (e_2), is to obtain as much S_3 as possible. Since e_1 is switched off, the majority of S_2 is converted into S_3. In the last phase the available components are transformed into the product (S_4) until the constraint in Eqn. (5) becomes active. In the last phase all enzymes are active but in such a way that the total amount of enzyme is not exceeded. Note also that reactants are almost consumed by the end of the process.

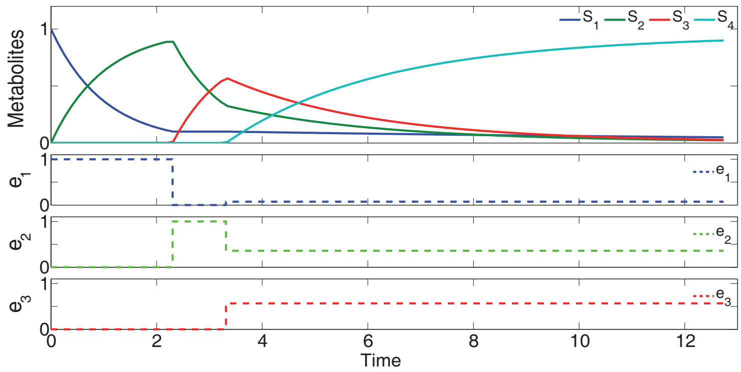

Figure S.2: **Metabolite dynamics and enzyme profiles corresponding to the minimum transition time in the three-step linear pathway (LPN3tt).** Metabolites and enzymes are expressed in concentration units and time in seconds (s).

Problem formulation and results for sub-case study of minimization of the time needed to obtain a certain amount of product (LPN3UB)

The objective in this case is the minimization of the time needed to reach a certain amount of product (Eqn. 7) when the substrate is consumed during the reaction process (unbuffered concentration of substrate, LPN3UB). The rest of the formulation remained unchanged.

$$J = t_f \tag{7}$$

The problem was initially solved with a multi-start of local solvers (Table S.6). And later, the obtained constrained NLP was solved with several nonlinear programing solvers. Results are reported in Table S.7.

For the sub-case, LPN3UB, where the objective is to minimize the time required to attain a certain product amount, the behavior of the system is shown in Figure S.3. The substrate is rapidly transformed into the first intermediate which is later transformed into the second intermediate and finally the product. In each step the corresponding enzyme is fully activated and the three steps last the same because all transformations are equally fast ($k_i = 1\forall i$). Note that since all the efforts are devoted to maximize production, final product amount is obtained in a shorter time ($t_f = 10.1\ s$).

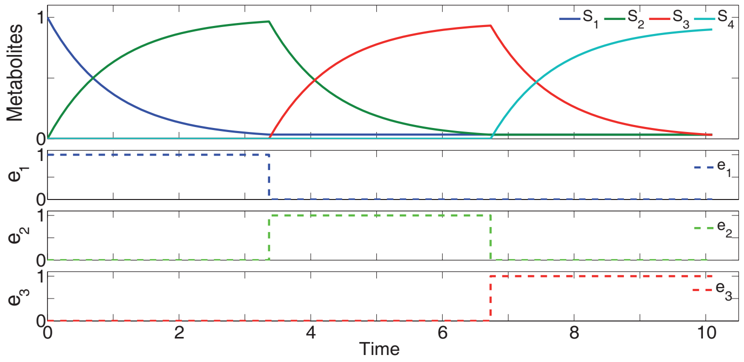

Figure S.3: **Metabolite dynamics and optimal enzyme profiles that minimize the time needed to reach a certain amount of product with unbuffered concentration of substrate (LPN3UB). Metabolites and enzymes are expressed in concentration units and time in seconds (s).**

Solution with a multi-start of local methods

The three single-objective cases considered (LPN3tt, LPN3UB, LPN3B) were initially solved with a multi-start of local solvers, this was used to i) analyze the properties of the different local solvers tested and ii) to analyze the convexity of the problem. Table S.6 presents a summary of the results whereas Figures S.4 present the histograms of the solutions achieved.

ACADO offers the best ratio of convergence to feasible solutions, however is the one reporting a larger percentage of convergence failures and a larger distribution of solutions thus reducing the convergence rate to the global. FSQP seems to be the best candidate in combination with CVP. In any case it seems clear that all methods converge to different solutions depending on the initial guess what calls for the use of global solvers.

Problem label	Solver statistics	FMINCON	SOLNP	FSQP	ACADO
LPN3tt	starts	1000	1000	1000	100
	failures %	0	0.1	0.1	11
	feasible %	15.4	15.1	15.1	86
	best	6.5676	6.5676	6.5676	6.5676
LPN3UB	starts	1000	1000	1000	100
	failures %	1.3	0	0	11
	feasible %	14.9	17	18	59
	best	10.099	10.099	10.099	10.099
LPN3B	starts	1000	1000	1000	100
	failures %	1.1	1.0	0	55
	feasible %	11.5	4.7	16.1	45
	best	4.222	4.222	4.222	4.222

Table S.6: **Summary of the solutions achieved for the LPN3 problems with the multi-start of local methods.** All methods arrive to the same best solution. However their individual behavior is different. "failures %" corresponds to the percentage of convergence failures reported by the solvers. "feasible %" corresponds to the percentage of feasible solutions found.

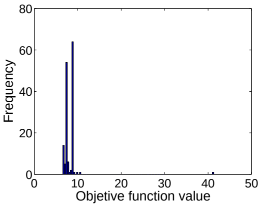

(a) FMINCON, LPN3tt

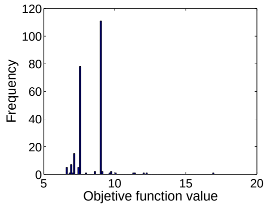

(b) SOLNP, LPN3tt

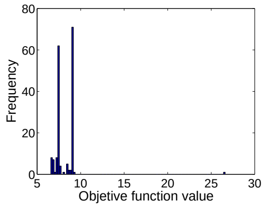

(c) FSQP, LPN3tt

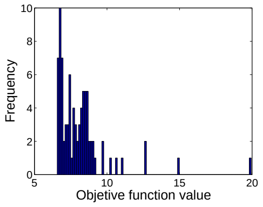

(d) ACADO, LPN3tt

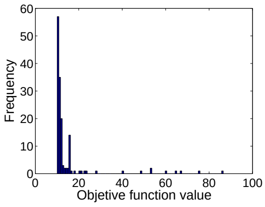

(e) FMINCON, LPN3UB

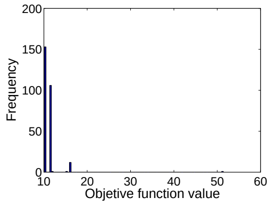

(f) SOLNP LPN3UB

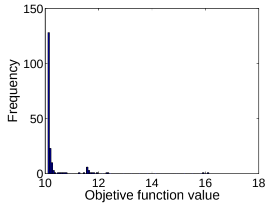

(g) FSQP, LPN3UB

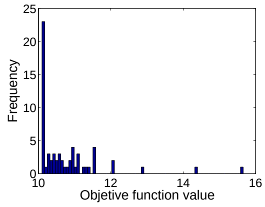

(h) ACADO, LPN3UB

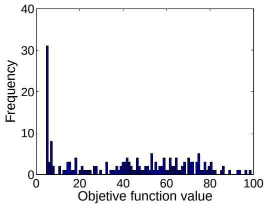

(i) FMINCON, LPN3B

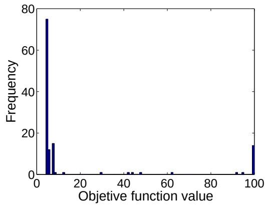

(j) SOLNP, LPN3B

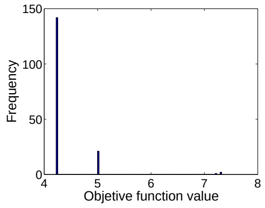

(k) FSQP, LPN3B

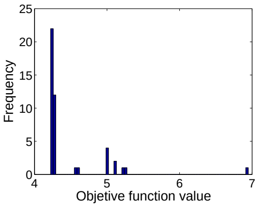

(l) ACADO, LPN3B

Figure S.4: **Histogram of solutions with different local solvers for the LPN3 examples.** Feasible solutions are shown against the frequency with which they were achieved. FMINCON and ACADO present larger distributions of solutions.

Solution with global and hybrid methods

In order to evaluate the performance of the global and hybrid methods, and due to their stochastic nature, 10 different runs were performed for each problem. Table S.7 presents a summary of the results achieved and Figures S.5 present the convergence curves for the best runs.

Problem label	Solver	Best	Worst	Mean	CPU time(s)
LPN3tt	eSS (FMINCON)	6.56765	6.56780	6.56768	36.5
	eSS (SOLNP)	6.56765	6.56769	6.56767	36.6
	**eSS(FSQP)**	**6.56765**	**6.56765**	**6.56765**	**33.2**
	SRES	6.95120	8.08152	7.80463	92.7
	hyb (SRES+FMINCON)	6.56789	6.82899	6.80214	47.1
	hyb (SRES+SOLNP)	6.56766	6.84409	6.75071	43.7
	hyb (SRES+FSQP)	6.56765	6.82899	6.74920	48.8
	DE	6.80393	7.00789	6.87412	101
	hyb (DE+FMINCON)	6.62149	6.82886	6.80675	60.3
	hyb (DE+SOLNP)	6.56767	6.91851	6.76363	58.3
	hyb (DE+FSQP)	6.56765	6.82668	6.80077	55.9
LPN3UB	eSS (FMINCON)	10.09908	10.09970	10.09913	24.5
	eSS (SOLNP)	10.09908	10.09975	10.09915	24.6
	**eSS(FSQP)**	**10.09908**	**10.09908**	**10.09908**	**27.4**
	SRES	10.42073	11.77762	10.99543	60.5
	hyb (SRES+FMINCON)	10.09909	11.68961	10.31823	35.2
	hyb (SRES+SOLNP)	10.09908	10.10146	10.09950	30.5
	hyb (SRES+FSQP)	10.09908	10.10085	10.31823	36.8
	DE	10.17363	10.71309	10.47218	134
	hyb (DE+FMINCON)	10.09908	10.29530	10.13981	66.1
	hyb (DE+SOLNP)	10.09908	10.10009	10.09927	63.7
	hyb (DE+FSQP)	10.09908	10.10146	10.09902	64.3
LPN3B	**eSS (FMINCON)**	**4.22210**	**4.22213**	**4.22212**	**24.5**
	eSS (SOLNP)	4.22213	4.22225	4.22214	24.3
	**eSS (FSQP)**	**4.22210**	**4.22214**	**4.22212**	**26.5**
	SRES	4.22704	4.61977	4.35825	49.9
	hyb (SRES+FMINCON)	4.22210	4.22214	4.22213	38.6
	hyb (SRES+SOLNP)	4.22210	4.22214	4.22212	34.9
	hyb (SRES+FSQP)	4.22210	4.22217	4.22213	31.1
	DE	4.23457	4.45597	4.30688	137
	hyb (DE+FMINCON)	4.22210	5.00285	4.45605	64.0
	hyb (DE+SOLNP)	4.22212	5.00252	4.30017	61.2
	hyb (DE+FSQP)	4.22210	4.22217	4.22214	50.9

Table S.7: **Summary of results achieved with global methods for the LPN3 examples.** Best corresponds to the cost function in the optimum found, worst corresponds to the worst value of the cost function in 10 runs of the method, mean corresponds to the mean of the 10 cost function values achieved, the CPU time in seconds corresponds to the mean of the 10 runs. Highlighted in gray is the overall best solution achieved.

Conclusions are similar in the three examples: eSS in combination with FSQP offers the best compromise robustness-computational effort, being able to arrive to the best known solution in around half a minute in all cases. As expected, SRES and DE approach the vicinity of the solution in reasonable times. However the

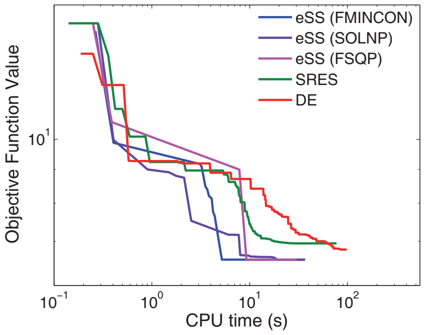

(a) LPN3tt

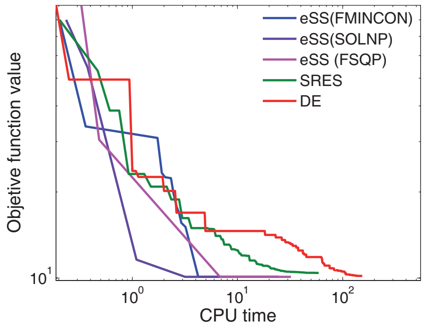

(b) LPN3UB

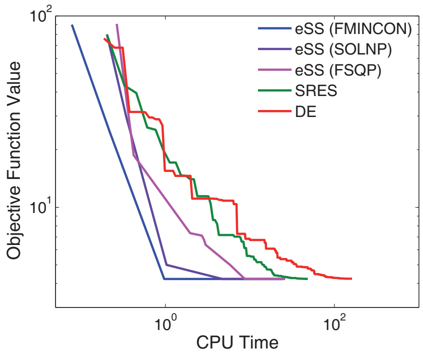

(c) LPN3B

Figure S.5: **Comparison of convergence curves for the LPN3 examples.** Figures present the evolution of the cost function with the computational effort. All methods are based on the use of populations. The initial population is different for each method therefore the first iterate may be different for all of them. In general, eSS is one order of magnitude faster than SRES or DE.

refining is too expensive, therefore the maximum number of iterations allowed for the optimization (Table S.5) is not enough to arrive to the best solution. In addition the distribution of the solutions achieved is rather significant specially for SRES. The combination of SRES and DE with local methods in sequential hybrids increases the chances of arriving to the global solution. In fact the hybrids with FSQP, allowed to arrive to the global solution in the three examples with reasonable computational costs but twice the CPU times required by eSS. It should be noted that the computational cost required by DE is usually larger than the ones required by SRES or eSS. This may be explained taking into account that current implementation uses death penalty to handle constraints. This approach guarantees feasibility of the solution, however it is more computationally demanding than the quadratic penalties used by eSS or the stochastic ranking approach used by SRES.

Five-step linear pathway with mass action kinetics (LPN5)

In addition to the examples presented in the main paper, we solve also an extra example performing the same analysis as in the other cases. This case is similar to the one addressed by Klipp et al. [2]. In that work

the authors computed the optimal enzyme switching points using a genetic algorithm and the final levels of enzyme so as to achieve a certain production. The optimal profiles correspond to a sequential activation of enzymes till the transition time is achieved. Oyarzun et al. [17] proposed a generalization of the problem, for a two enzymes case, where not only the switching times but the levels of the enzymes were considered as decision variables. Here, we consider this second formulation for the case of a five-step linear pathway performing an analysis of the multimodal nature of the problem and checking different nonlinear programing solvers to find the most adequate one.

Five-step linear pathway with mass action kinetics (LPN5)

The pathway converts the substrate (S_1) into the product (S_6) using five steps (as shown in Figure S.6), each one catalyzed by a specific enzyme (e_i). The aim of this problem is to minimize the transition time. The transition time is esteemed as the time required to attain a certain conversion of the substrate into the product by means of the intermediates, 90% in this case [16, 17]. The model has arbitrary units.

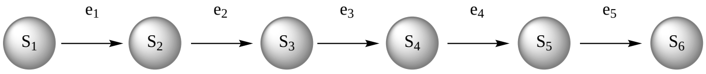

Figure S.6: **Schematic representation of the five-step linear pathway (LPN5).** Metabolites and enzymes are expressed in arbitrary units.

The mathematical statement is as follows. Find $\mathbf{e}(t)$ over $t \in [t_o, t_f]$ to minimize the transition time to reach a fixed amount of product:

$$\tau = \int_{t_0}^{t_f} (1 - S_6) dt \tag{8}$$

Subject to the system dynamics:

$$\frac{d\mathbf{S}}{dt} = \mathbf{N}\boldsymbol{\upsilon} \tag{9}$$

Where N:

$$\mathbf{N} = \begin{bmatrix} -1 & 0 & 0 & 0 & 0 \\ 1 & -1 & 0 & 0 & 0 \\ 0 & 1 & -1 & 0 & 0 \\ 0 & 0 & 1 & -1 & 0 \\ 0 & 0 & 0 & 1 & -1 \\ 0 & 0 & 0 & 0 & 1 \end{bmatrix}$$

And:

$$\upsilon_i = k_i \cdot S_i \cdot e_i \tag{10}$$

With the following end point constraints:

$$S_6(t_f) = P(t_f) \tag{11}$$

And the following path constraint:

$$\sum_{i=1}^{5} e_i \leq E_T \tag{12}$$

where S_1 corresponds to the substrate, $S_2 - S_5$ to the intermediate metabolites and S_6 to the product; e_i correspond to the enzymes and $E_T = 1$, $k_i = 1$, $S_1(t_0) = 1$, $S_i(t_0) = 0$ for $i = 1, 2, 3, 4, 5$, $P(t_f) = 0.9$.

The dynamic optimization problem is transformed into a NLP assuming a step-wise approximation of the enzymes profiles. The total number of decision variables is 30 (5 enzymes· 5 steps+ 5 switching times) and 6 constraints. The problem was initially solved with a multi-start of local solvers (Table S.8) This problem is particularly complicated, only eSS and the sequential hybrid of SRES with SOLNP were able to arrive to the optimal solution $\tau = 14.4$. Results obtained by other methods are summarized in Table S.9.

Optimal profiles (Figure S.7) are similar to those reported by Klipp et al. [2], despite the differences in problem formulation. The Figure shows that the reaction conversion proceeds in two phases. In the first phase (during around 10 s) the reactions operate in sequence, following a bang-bang profile of activation of enzymes $e_1 - e_4$, in such a way that all intermediates are produced and partially consumed and no product is generated. In the second phase, enzymes $e_3 - e_5$ are partially activated so as to produce the desired amount of product. Note that from a biological point of view the use of e_3 and e_4 in the last phase of the process may be disadvantageous for the cell and therefore the solution may not be realistic. Remark that this may be solved if the enzyme consumption is incorporated in the objective, i.e. if the minimization of the enzyme activity is also pursued.

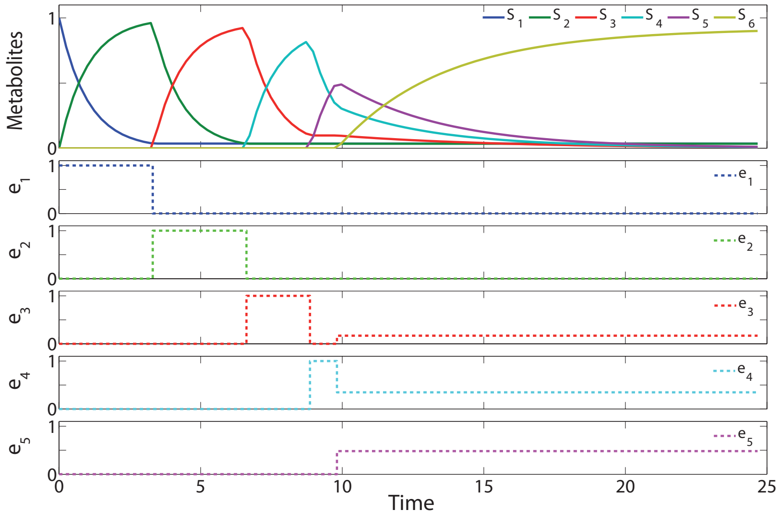

Figure S.7: **Metabolite dynamics and enzyme activation profiles that minimize the transition time to achieve a certain amount of product in a five-step linear pathway (LPN5).** Metabolites and enzymes are expressed in arbitrary units.

Solution with a multi-start of local methods

As for the previous examples, a multi-start of local methods was used to analyze the possible multimodality of the problem. Table S.8 summarizes the results. For this example, the best solution was achieved by

ACADO toolkit. This method reported the largest percentage of success in finding feasible solutions but also the largest percentage of convergence failures. The percentages of success in finding feasible solutions are really low for all methods in combination with the CVP approach. To analyze the results in more detail, Figures S.8 show the histograms of the feasible solutions achieved. All methods end up in large distributions of the solutions. It should be noted that for most of the methods some of the solutions achieved correspond to more than 100% absolute error with respect to the best found, this reflecting the complexity of the problem.

Solver	FMINCON	SOLNP	FSQP	ACADO
starts	2000	1000	1000	100
failures %	0.05	0.3	0	19
feasible %	4.75	2.3	1.1	81
best	14.441	14.443	14.600	14.435

Table S.8: **Summary of the solutions achieved for the LPN5 example with the multi-start of local methods.** ACADO results in the best solution whereas the CVP in combination with FSQP results in the worst value.

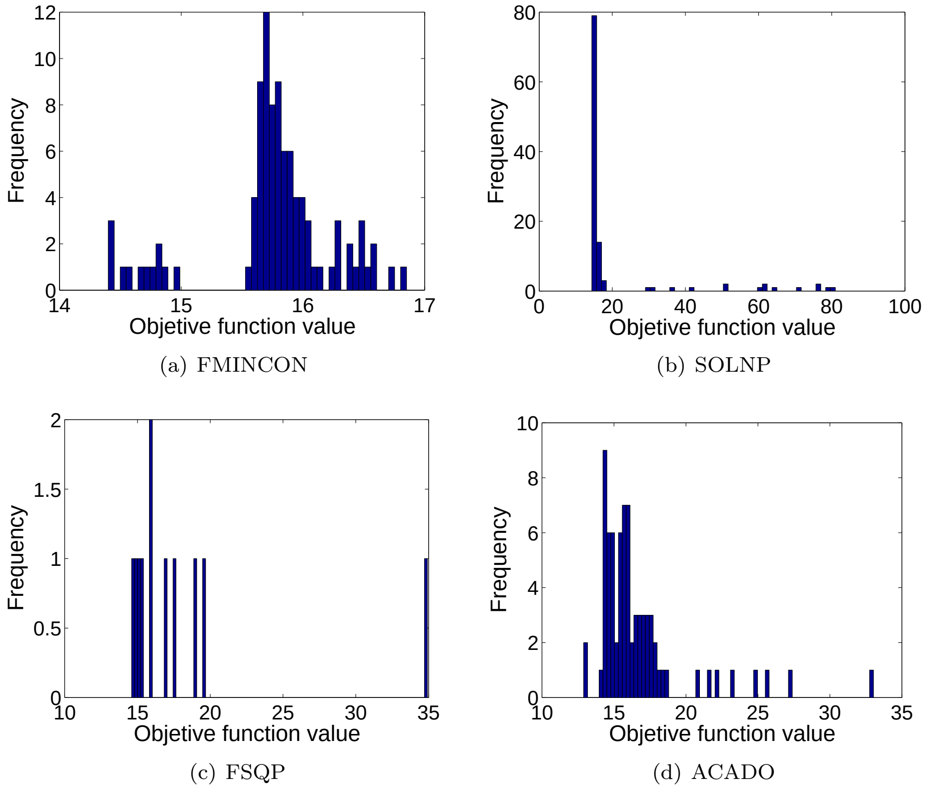

(a) FMINCON (b) SOLNP

(c) FSQP (d) ACADO

Figure S.8: **Histogram of solutions with different local solvers for the LPN5 example.** Feasible solutions are shown against the frequency with which they were achieved. FMINCON and ACADO are the most successful in this example. FMINCON presents a distribution of solutions close to the best found by ACADO. ACADO presents a larger distribution of solutions but reports the best solution.

Solution with global and hybrid methods

The multi-start of local methods revealed the necessity of using global optimization to deal with this problem. Solutions achieved by the different global methods considered in this work are summarized in Table S.9.

Solver	Best	Worst	Mean	CPU time(s)
eSS (FMINCON)	14.43567	14.74693	14.49364	92.2
eSS (SOLNP)	14.43567	14.68277	14.46029	93.3
**eSS (FSQP)**	**14.43567**	**14.57931**	**14.45505**	**102**
SRES	15.61308	17.14946	16.93167	951
hyb (SRES+FMINCON)	15.35850	17.15250	16.22782	177
hyb (SRES+SOLNP)	14.43567	15.36097	15.19905	158
hyb (SRES+FSQP)	14.55342	15.36353	15.07467	135
DE	15.99794	17.90227	17.06582	1118
hyb (DE+FMINCON)	14.74083	16.61492	15.43213	81.9
hyb (DE+SOLNP)	14.68276	15.53565	15.00343	108
hyb (DE+FSQP)	14.60562	17.21147	15.12188	117

Table S.9: **Summary of results achieved with global methods for the LPN5 example.** Best corresponds to the cost function in the optimum found, worst corresponds to the worst value of the cost function in 10 runs of the method, mean corresponds to the mean of the 10 cost function values achieved, the CPU time in seconds corresponds to the mean of the 10 runs. Highlighted in gray is the overall best solution achieved.

The optimum value (14.3567) was obtained with eSS combined with the three different local solvers. The best worst and mean values were achieved with FSQP. Therefore this combination is considered to be the best in this example, even though the required computational time is 10% longer. It should be noted that only the hybrid of SRES and FSQP was able to converge to the global solution requiring 50% more computational time. Despite the hybrids with DE performed better in average, the fact is that they were not able to converge to the optimal solution.

Figure S.9 presents the convergence curves for each method. From the curves it is clear that eSS provides the fastest converge rate. SRES and DE seem to need even more iterations (the maximum was 3500) to refine the solutions, this is particularly clear for DE whose convergence curve does not present the typical flat area at the end of the convergence curve.

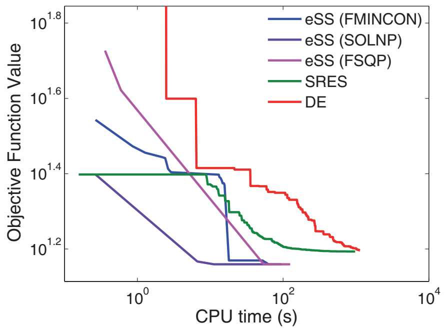

Figure S.9: **Comparison of convergence curves for the LPN5 example.** eSS arrives independently of the local method used. Global solvers were not able to arrive to the global solution in the allowed maximum number of iterations. eSS is at least one order of magnitude faster than SRES or DE.

Four-step linear pathway with Michaelis-Menten kinetics (LPDN4)

In [3] two single-objective situations were considered for a four-step linear pathway: LPN4 corresponds to the case were activation of enzymes is assumed to be instantaneous and LPDN4 incorporates enzyme activation dynamics. In this section problem formulation and solution to the instantaneous case is presented, besides that an analysis of the multimodal nature of the single-objective problem for both examples was preformed. Also here we face the solution of LPN4 and LPDN4 with different global and hybrid methods.

Instantaneous enzyme activation-deactivation (sub-case LPN4)

This example was originally considered by Oyarzun et al. [3]. The authors demonstrated the existence of solution by means of the Pontryagin's maximum principle and solved the problem using a local SQP based method (fmincon as included in the MATLAB Optimization Toolbox). The pathway (Figure S.10) consists in four enzymatic reactions catalyzed by a specific enzyme (e_i) where S_1 corresponds to the substrate, $S_2 - S_3$ to the intermediate metabolites and S_4 to the product. The objective is to minimize the sum of the time needed to reach a given steady state while minimizing enzyme consumption.

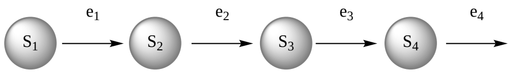

Figure S.10: **Schematic representation of a four-step linear pathway.** It consists in four enzymatic reactions, where the substrate (S_1) is converted into the product (S_4).

This mathematically reads:

Find $\mathbf{e}$ over $t \in [t_o, t_f]$ to minimize:

$$J = \int_{t_0}^{t_f} (1 + \sum_{i=1}^{n=4} \mathbf{e}_i) dt \quad (13)$$

Subject to the system dynamics:

$$\frac{d\mathbf{S}}{dt} = \mathbf{N}\boldsymbol{v} \tag{14}$$

with:

$$\mathbf{N} = \begin{bmatrix} -1 & 0 & 0 & 0 \\ 1 & -1 & 0 & 0 \\ 0 & 1 & -1 & 0 \\ 0 & 0 & 1 & -1 \end{bmatrix}$$

And:

$$v_i = \frac{k_{cati} \cdot S_i}{K_M + S_i} \cdot e_i \tag{15}$$

With the following end point constraints:

$$v_i(t_f) = V \tag{16}$$

And the following next path constraint:

$$0 \leq \sum_{i=1}^{4} e_i \leq E_T \quad \& \quad e_i \geq 0 \forall i \tag{17}$$

With: $E_T = 1\ mM$, $k_{cat1} = 1\ s^{-1}$, $k_{cat2} = 2\ s^{-1}$, $k_{cat3} = 4\ s^{-1}$, $k_{cat4} = 3\ s^{-1}$, $K_M = 1\ s^{-1}$, $V = 0.2$ mM/s, $S_1(t_0) = 5\ mM$, $S_i(t_0) = 0$ for $i = 2, 3, 4$.

In this case, the CVP approach transforms the dynamic optimization problem into a NLP with 20 decision variables (4 enzymes·4 steps+4 switching times). The problem was initially solved with a multi-start of local solvers (Table S.10) and with several nonlinear programing solvers (Table S.11). The optimal solution ($J^* = 4.6562$) was achieved with eSS. It should be noted that this result slightly improves previous published results ($J^* = 4.7401$) [3, 18].

The metabolite dynamics and optimal enzyme profiles are shown in Figure S.11. The optimal solution corresponds with a bang-bang profile where each enzyme is activated, respecting the amount of resources available (Eqn. 17), following a temporal sequence that matches with the pathway topology. At final time a certain amount of enzymes is required so as to satisfy the end-point constraint (Eqn. 16).

Since the consumption of S_1 is slower than for the intermediates and the product, a significant amount of e_1 is required so as to achieve the necessary amount of S_2. Thus e_1 is activated more than the 50% of the process duration. Afterwards e_2, e_3 and e_4 are activated in sequence so as to produce the required amounts of S_3 and S_4. At the end of the reaction the available enzymes are switched on so as to achieve the requested flux level (Eqn. 16).

Solution with a multi-start of local methods

Both examples were firstly solved using a multi-start of the local methods. Unfortunately in these cases the rate of success was very low. FSQP and SOLNP were not able to find feasible solutions from any of the initial points in the multi-start and ACADO was reporting a substantial percentage of convergence failures. This reveals that the feasible area is small and therefore finding the optimal solution is complicated.

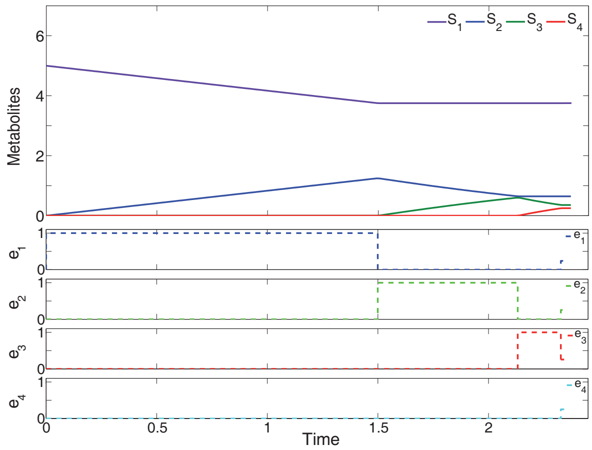

Figure S.11: **Metabolite dynamics and enzyme activation profiles that minimize time needed to reach a given steady state and the corresponding enzyme consumption (LPN4).** Enzyme and metabolite concentrations are expressed in concentration units (mM) and time in seconds.

Problem label	Solver	FMINCON	ACADO
LPN4	starts	2000	100
	failures %	0	65
	feasible %	0.9	23
	best	4.9487	4.8275
LPDN4	starts	2000	100
	failures %	0	87
	feasible %	0	13
	best	-	6.1589

Table S.10: **Summary of the solutions achieved for the LPN4 problems with the multi-start of local methods.** The best solution was obtained by ACADO in both cases. Percentage of feasible solutions is very low as compared to previous examples.

Solution with global and hybrid methods

Table S.11 presents the results achieved by global and hybrid methods. DE was not able to find a feasible solution in the allowed maximum number of iterations. The best values were obtained with eSS and FMINCON as local solver. It should be remarked that those values had not been achieved with the multi-start of local methods. The hybrid of SRES with fmincon arrived to the vicinity of the solution but with twice the computational cost required by eSS to converge.

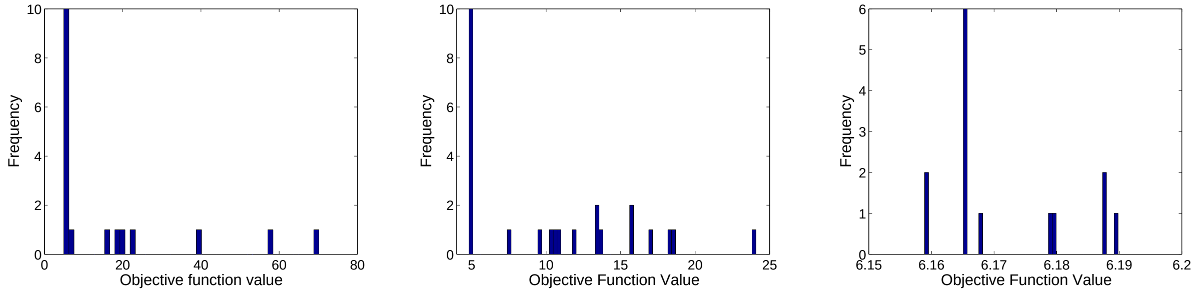

(a) FMINCON LPN4 (b) ACADO LPN4 (c) ACADO LPDN4

Figure S.12: **Histogram of solutions with different local solvers for the LPN4 examples.** Feasible solutions are shown against the frequency with which they were achieved. The number of feasible solutions is very low and for the LPN4 the dispersion of the solutions is rather significant (a and b). Note that for the LPDN4 case (c) only 13 out of 100 runs converged to feasible solutions all of them close to the best found.

Problem label	Solver	Best	Worst	Mean	CPU time(s)
LPN4	**eSS (FMINCON)**	**4.65624**	**4.65671**	**4.65639**	**157**
	SRES	5.37667	21.57404	9.07416	307
	hyb (SRES+FMINCON)	4.65692	4.94926	4.71339	279
LPDN4	**eSS (FMINCON)**	**6.15438**	**6.19895**	**6.17518**	**169**
	SRES	6.56442	8.17242	7.29639	616
	hyb (SRES+FMINCON)	6.16186	6.54851	6.21896	374

Table S.11: **Summary of results achieved with global methods for the LPN4 examples.** eSS found the best solution in both cases in less than 3 min of computational cost. SRES stopped far from the optimum in both cases. They sequential hybrid arrived to vicinity of the global in all runs but the dispersion of the solutions and the computational cost are larger than with eSS.

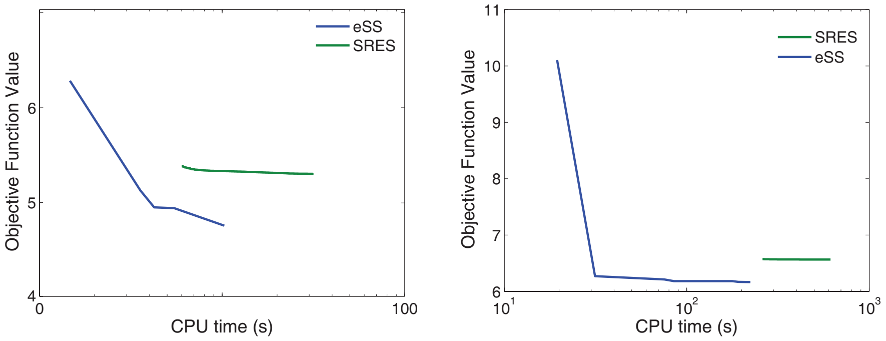

(a) LPN4 (b) LPDN4

Figure S.13: **Comparison of convergence curves for the LPN4 examples.** SRES was not able to arrive to the global solution in the allowed maximum number of iterations. eSS is at least one order of magnitude faster than SRES.

Glycolysis inspired network (GDB)

In [5] the authors present the network with instantaneous enzyme activation for two different situations when the substrate is consumed (GUB) and when the substrate is buffered (GB), not consumed during the process. In our example we extend that formulation by incorporating the enzyme dynamics, besides the single-objective example presented in the main text (GDUB). Here we present the situation when the substrate is consumed during the process (GDB). Additionally a detailed analysis of the multimodal nature of the problem was performed for all single-objective situations. Different global and hybrid methods were tested to find the most efficient one.

Instantaneous enzyme activation (sub-cases GUB and GB)

This formulation was considered by Bartl et al. [5] with the Pontryagin's maximum principle. The pathway (Figure S.14) consists of four enzymatic reactions with one branch where S_1 corresponds to the substrate, $S_2 - S_4$ to the intermediate metabolites and S_5 to the product. The hypothesis in this problem is that the pathway activation minimizes the time needed to transform the substrate (S_1) into a fixed amount of product (S_5). Two different situations were considered when the substrate is, at least partially, consumed, this situation corresponds with $\mathbf{N}_{UB}$ (GUB) and where the substrate is buffered, this situation is represented by $\mathbf{N}_B$ (GB).

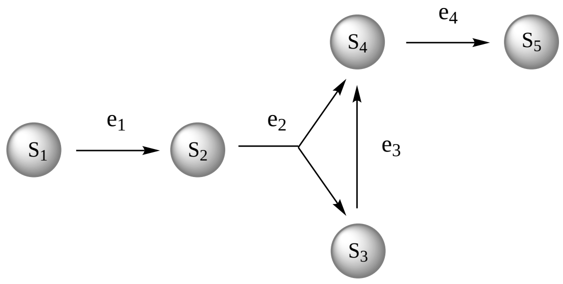

Figure S.14: **Schematic representation of a Glycolysis inspired pathway.** Metabolites and enzymes are expressed in concentration units and time in seconds (s).

The activation profiles may be acquired by computing $\mathbf{e}$ over $t \in [t_o, t_f]$ to minimize the cost function (18).

$$J = t_f \tag{18}$$

Subject to the system dynamics:

$$\frac{d\mathbf{S}}{dt} = \mathbf{N}\boldsymbol{v} \tag{19}$$

Where:

$$v_i = \frac{k_{cati} \cdot S_i}{K_M + S_i} \cdot e_i \tag{20}$$

With:

$$\mathbf{N}_{UB} = \begin{bmatrix} -1 & 0 & 0 & 0 \\ 1 & -1 & 0 & 0 \\ 0 & 1 & -1 & 0 \\ 0 & 1 & 1 & -1 \\ 0 & 0 & 0 & 1 \end{bmatrix} \qquad \mathbf{N}_{B} = \begin{bmatrix} 0 & 0 & 0 & 0 \\ 1 & -1 & 0 & 0 \\ 0 & 1 & -1 & 0 \\ 0 & 1 & 1 & -1 \\ 0 & 0 & 0 & 1 \end{bmatrix}$$

And the following end point constraints:

$$S_5(t_f) = P(t_f) \tag{21}$$

And the following next path constraint:

$$\sum_{i=1}^{4} e_i \leq E_T \tag{22}$$

With $E_T = 1\ mM$, $P(t_f) = 0.75\ mM$, $k_{cati} = 1\ s^{-1}$, $K_M = 1\ s^{-1}$, $S_1(t_0) = 1\ mM$, $S_i(t_0) = 0$ for $i = 2, 3, 4, 5$.

The problems were solved with a multi-start of local solvers (Table S.12). Later, the problem was solved with several nonlinear programing solvers. Results are reported in Table S.13. The total number of decision variables in the NLP is 20 (4 enzymes · 4 steps + 4 switching times) and 5 constraints. The optimal solution for $\mathbf{N}_{UB}$ (GUB) is $J^* = 7.9$ and for $\mathbf{N}_B$ (GB), $J^* = 6.9$, in both cases the best result was obtained using eSS, further details may be found in Table S.12. The corresponding metabolite and enzyme concentrations are shown in Figures 10 and 11 respectively. In both cases the optimal enzyme activation corresponds with a bang-bang profile where each enzyme is activated following the pathway topology. Even though the optimal times were not reported and thus can not be directly compared, the optimal enzyme profiles obtained are in good agreement with the ones reported by Bartl et al. [5].

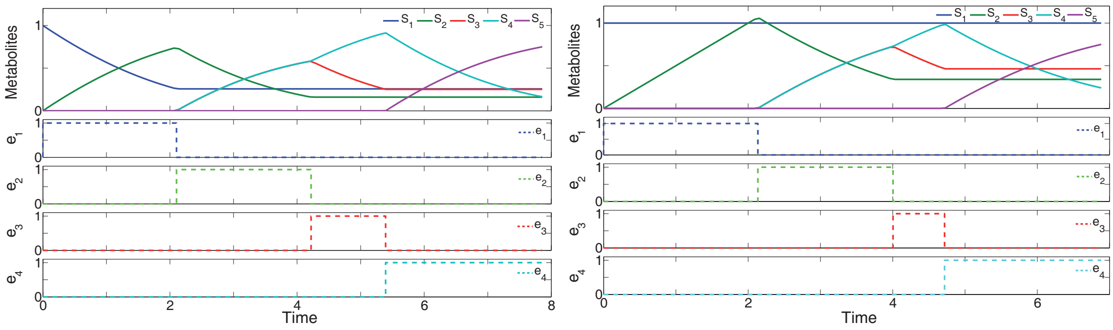

(a) GUB (b) GB

Figure S.15: **Optimal profiles for the Glycolysis inspired pathway for both situations considered**. Metabolites and enzymes are expressed in concentration units and time in seconds (s).

This *"just-in-time"* enzyme activation profile was also found in previous examples, inducing the production of the corresponding metabolites in sequence. Note, however, that the introduction of a branch in the network results in a different intermediates production profile (Figure S.15). In fact S_3 and S_4 appear simultaneously since the activation of e_2 contributes to the formation of both intermediates, the activation

of e_3 contributes to increase the production of S_4 and finally e_4 is active till the desired amount of S_5 is produced (Eqn. 21). This response is similar in the unbuffered and the buffered case, but the process is slightly faster in the buffered case.

With enzyme dynamics and substrate consumption (sub-case GDUB)

The single-objective problem formulation presented in the main text is also solved for the situation where substrate is consumed, represented by matrix $\mathbf{N}_{UB}$

$$\mathbf{N}_{UB} = \begin{bmatrix} -1 & 0 & 0 & 0 \\ 1 & -1 & 0 & 0 \\ 0 & 1 & -1 & 0 \\ 0 & 1 & 1 & -1 \\ 0 & 0 & 0 & 1 \end{bmatrix}$$

As in the other cases, the problem was solved with a multi-start of local solvers (Table S.12). Later, the problem was solved with several nonlinear programing solvers. Results are reported in Table S.13. The optimal value is $J^* = 10.5$ and was obtained in lees than 50 s of CPU time. The corresponding optimal enzyme activation profiles are shown in Figure S.16. Again the optimal profiles for the expression rate follow a switching pattern that matches with the pathway topology leading to enzyme profiles that follow a sequential activation profile. Note that the time needed for the process is slightly higher that in the inexhaustible case.

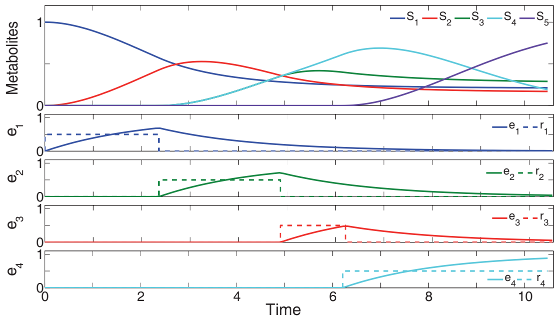

Figure S.16: **Metabolite and enzyme dynamics for the Glycolysis inspired pathway coupled with the enzyme dynamics in the unbuffered situation (GDUB)**. This profiles were obtained with eSS and FSQP as local solver. Enzyme and metabolite concentrations are expressed in concentration units (mM) and time in seconds (s).

Solution with a multi-start of local methods

All problems were solved by means of a multi-start of local methods. Results are summarized in Table S.12. For the GUB case, the CVP approach with FMINCON or FSQP report the best result, whereas ACADO obtains the worst value despite the high ratio of convergence to feasible solutions. For the GB ,GDUB and GDUB cases, ACADO reports the best result. It should be noted that the CVP in combination with local methods is in general able to converge to feasible solutions in less than the 5% of the runs. Multiple shooting

is more successful although it reports a substantial percentage (between the 27 and the 50) of convergence failures.

Problem label	Solver	FMINCON	SOLNP	FSQP	ACADO
GUB	starts	1000	1000	1000	100
	failures %	0	0.3	0	30
	feasible%	4.7	1.8	4.7	64
	best	7.8578	7.8579	7.8578	7.8589
GB	starts	1000	1000	1000	100
	failures%	0	3.5	0	50
	feasible%	2.7	26.5	1.8	40
	best	6.8844	6.8937	6.8937	6.8837
GDUB	starts	1000	1000	1000	100
	failures%	0	0.4	0	27
	feasible%	3.6	0.8	3.9	57
	best	10.4781	10.8318	10.4781	10.478
GDB	starts	1000	1000	1000	100
	failures%	0	0.5	0	37
	feasible%	0.3	0.4	4.1	53
	best	9.6125	9.7788	9.4889	9.4887

Table S.12: **Summary of the solutions achieved for the glycolysis problems with the multi-start of local methods.** The best solution was obtained by the CVP approach with FMINCON or FSQP for GUB and by ACADO for GB, GDUB and GDB.

Histograms of feasible solutions are shown in Figures S.17 showing how for some methods/problems the distribution of the solutions is significant. It should be noted that, differently to previous examples, the rate of convergence to the vicinity of the best solution is higher in these examples (left bar is the longest in all cases).

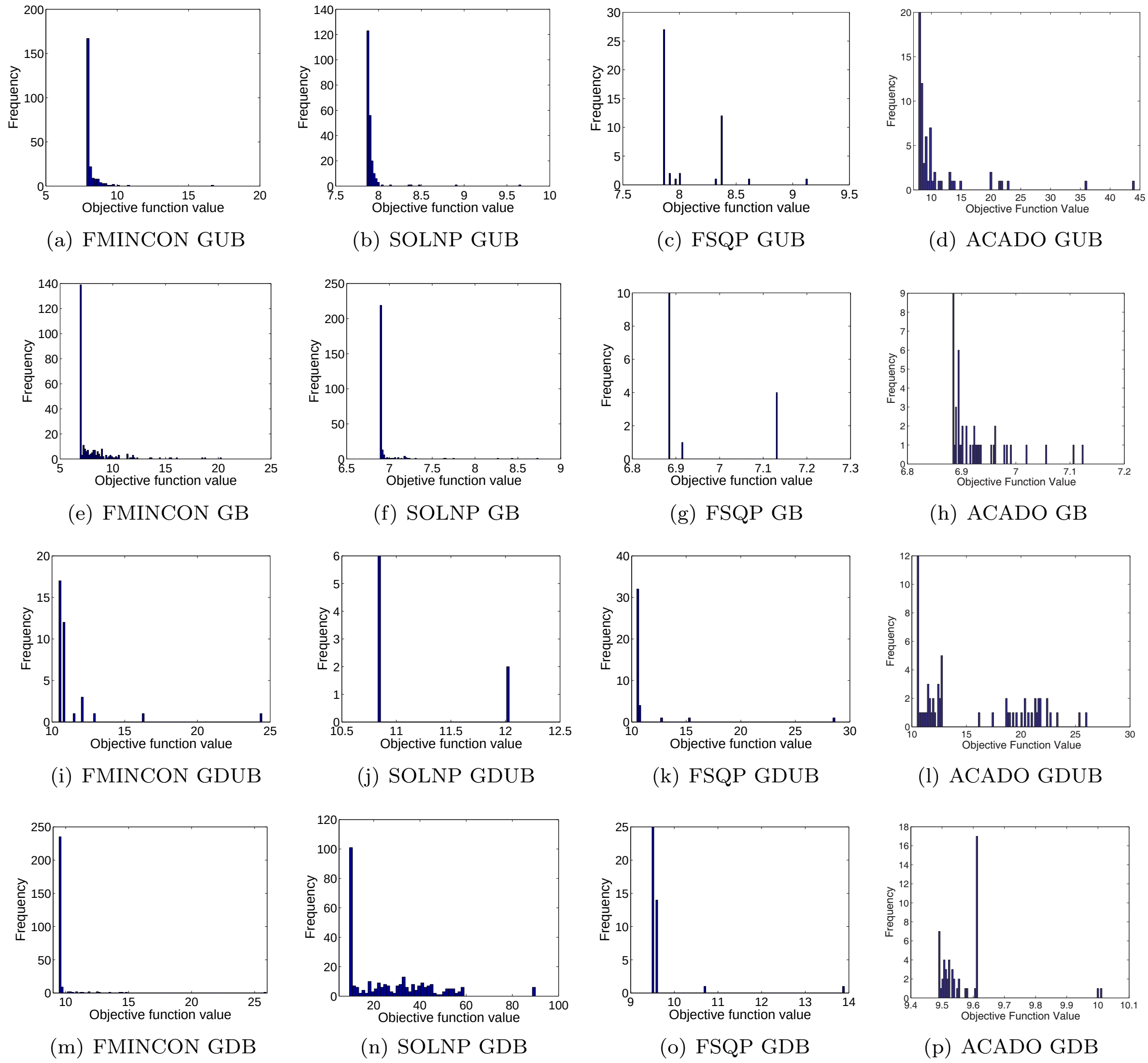

Figure S.17: **Histogram of feasible solutions with different local solvers for the glycolysis examples.** The presence of distribution of solutions call for the use of global optimizers.

Solution with global and hybrid methods

Optimization results are summarized in Table S.13. eSS combined with either FMINCON or FSQP provides the best results in all cases and with almost no dispersion on the results. For the case GDB the optimal solution had not been found with the multi-start of local methods. Neither DE nor SRES were able to converge to the global solution in the allowed number of iterations. In any case SRES performs better than DE basically due to the stochastic ranking of the constraints. Sequential hybrids were able to converge to the global solution at least once, but the computational cost is twice or three times the cpu time required by eSS. In general hybrids with DE are more robust than those with SRES but at the expense of longer computational costs.

Problem label	Solver	Best	Worst	Mean	CPU time(s)
GUB	**eSS (FMINCON)**	**7.85778**	**7.85778**	**7.85778**	**40.7**
	eSS (SOLNP)	7.85778	7.85781	7.85779	41.4
	**eSS (FSQP)**	**7.85778**	**7.85778**	**7.85778**	**40.7**
	SRES	8.13481	8.69189	8.44731	180
	hyb (SRES+FMINCON)	7.85778	8.37280	7.90957	85.6
	hyb (SRES+SOLNP)	7.85778	7.86032	7.85836	94.5
	hyb (SRES+FSQP)	7.85778	8.37358	7.90936	77.2
	DE	8.72122	10.3803	9.52464	425
	hyb (DE+FMINCON)	7.85778	8.37537	8.01265	169
	hyb (DE+SOLNP)	7.85778	8.37358	8.01233	95.6
	hyb (DE+FSQP)	7.85778	8.37537	7.96130	105
GB	**eSS (FMINCON)**	**6.88366**	**6.88367**	**6.88366**	**41.0**
	eSS (SOLNP)	6.88367	6.88367	6.88367	40.9
	**eSS (FSQP)**	**6.88366**	**6.88367**	**6.88366**	**29.3**
	SRES	7.05783	7.45541	7.23051	169
	hyb (SRES+FMINCON)	6.88371	7.13313	6.98247	82.6
	hyb (SRES+SOLNP)	6.88372	7.13348	7.00739	81.7
	hyb (SRES+FSQP)	6.88367	6.88367	6.88367	83.6
	DE	7.62285	8.57032	7.96773	556
	hyb (DE+FMINCON)	6.88367	7.13339	6.97025	123
	hyb (DE+SOLNP)	6.88368	7.13323	6.95858	121
	hyb (DE+FSQP)	6.88367	7.13318	6.90862	124
GDUB	**eSS (FMINCON)**	**10.47813**	**10.47819**	**10.47814**	**50.5**
	eSS (SOLNP)	10.47814	10.83098	10.53691	67.7
	**eSS (FSQP)**	**10.47813**	**10.47813**	**10.47813**	**42.4**
	SRES	10.62624	11.56653	10.95767	329
	hyb (SRES+FMINCON)	10.47820	11.26087	10.72655	100
	hyb (SRES+SOLNP)	10.47816	10.83213	10.69042	106
	hyb (SRES+FSQP)	10.47813	10.83186	10.53709	86.7
	DE	11.61289	13.08953	12.26030	571
	hyb (DE+FMINCON)	10.47811	11.46896	10.64330	162
	hyb (DE+SOLNP)	10.47813	10.83192	10.53713	157
	hyb (DE+FSQP)	10.47813	10.47813	10.47813	144
GDB	**eSS (FMINCON)**	**9.48873**	**9.48889**	**9.48887**	**40.6**
	eSS (SOLNP)	9.48873	9.61240	9.51354	80.5
	**FSQP)**	**9.48873**	**9.48873**	**9.48873**	**41.01**
	SRES	9.62507	10.7444	9.95951	179
	hyb (SRES+FMINCON)	9.48893	9.61274	9.58784	76.5
	hyb (SRES+SOLNP)	9.48895	9.61277	9.56313	75.9
	hyb (SRES+FSQP)	9.48873	9.61241	9.52597	84.1
	DE	10.0746	11.5562	10.8364	647
	hyb (DE+FMINCON)	9.48895	9.61314	9.58991	156
	hyb (DE+SOLNP)	9.48891	9.61265	9.58991	157
	hyb (DE+FSQP)	9.48895	9.612445	9.56307	136

Table S.13: **Summary of results achieved with global methods for the glycolysis related examples.** eSS found the best solution in all cases in around 40 s of computational cost.

Figures S.18 present the convergence curves of the different methods in the solution of the four cases considered. Conclusions are similar to the ones obtained in previous examples. eSS is at least one order of magnitude faster than stochastic global methods. The death penalty approach makes DE to be the slowest of the approaches visiting many unfeasible solutions and thus slowing down the convergence.

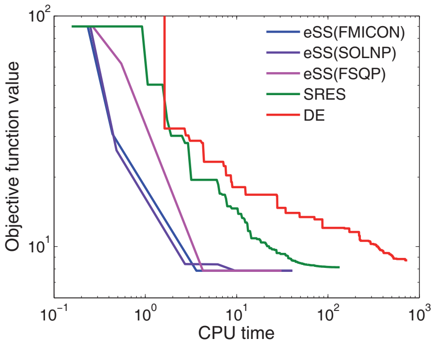

(a) GUB

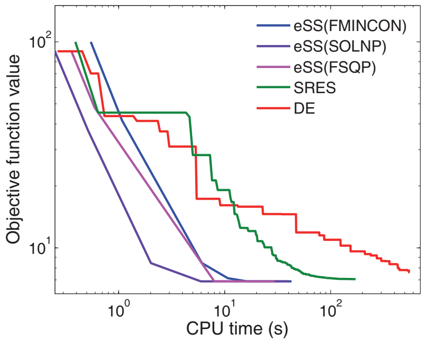

(b) GB

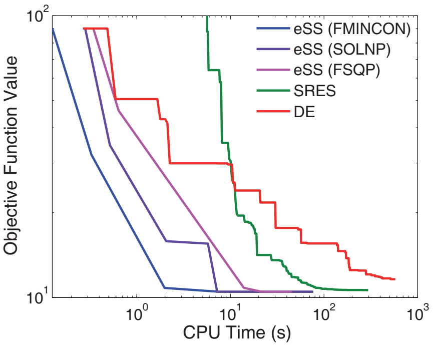

(c) GDUB

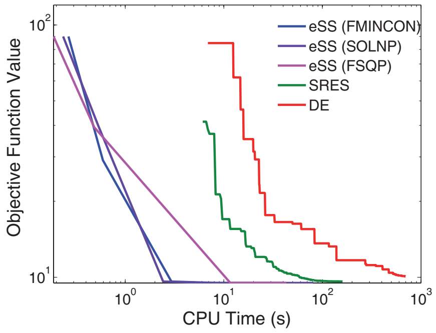

(d) GDB

Figure S.18: **Comparison of convergence curves for the glycolysis related examples.**

Central metabolism of *Saccharomyces cerevisiae* during diauxic shift (SC)

In this section we analyze the multimodal nature of the problem. Beside of this we solve the single-objective problem with different hybrid and global solvers using two different approaches i) a step-wise approximation with 120 constant elements for each enzyme (i.e. 721 decision variables) and ii) a piecewise linear approximation with 8 variable length linear elements (i.e. 55 decision variables). Additionally for the multi-objective case, completed optimization profiles are depicted here.

Solution with a multi-start of local methods

The optimization problem was solved using a multi-start of local methods. Table S.14 summarizes the results. For this problem the best solution was achieved by SOLNP even though its ratio of success was very low. The percentage of convergence failures in this examples was significant for all methods. In addition the convergence to feasible solutions was rather complicated indicating that the feasible area is small, making it difficult to find the global maximum.

Solver	FMINCON	SOLNP	FSQP	ACADO
starts	1000	1000	1000	100
failures %	66	20.8	98.7	55
feasible%	1.2	0.6	1.3	45
best	91.3652	94.59276	89.8895	94.236

Table S.14: **Summary of the solutions achieved for the *Saccharomyces cerevisiae* case with a multi-start of local methods.**

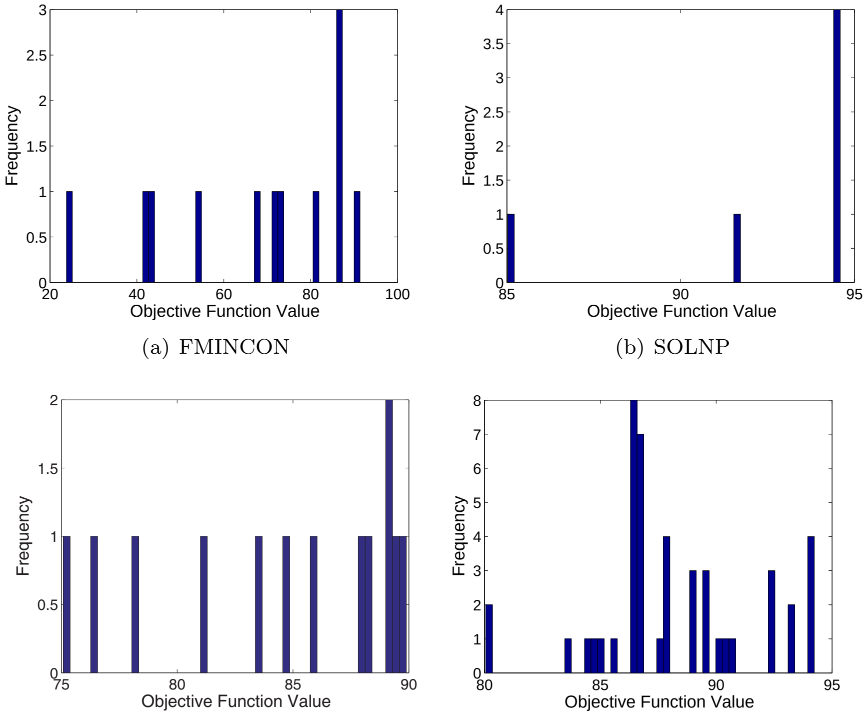

(a) FMINCON (b) SOLNP

(c) FSQP (d) ACADO

Figure S.19: **Histogram of feasible solutions with different local solvers for the *S. cerevisiae* example.**

Solution with global and hybrid methods

As commented in the main text the solution of this problem was addressed with two types of CVP approximations: in one hand, a piece-wise linear interpolation with 8 time varying elements and in the other hand (PWL-v), with a step-wise interpolation with 120 constant length elements (PWC).

Table S.15 presents the results obtained. Showing how eSS was the method reporting the best value, which is better than the one found by means of the multi-start. It should be noted that the best value was achieved with the PWL-v interpolation of the enzymes. The use of PWC profiles results in suboptimal noisy optimal profiles (see Figure S.21) and the computational effort is one order of magnitude higher. It should be noted, however, that the dispersion of the solutions out of the 10 runs performed, is significantly lower in the case with PWC approximation, this is because the introduction of switching times as decision variables increases the multimodality of the problem. As an overall result, the mean values achieved by the use of PWC approximation are better than the ones obtained with PWL-v.

SRES was not able to report feasible solutions and DE was not able to refine the solution in the maximum allowed effort. The hybrids performed slightly better however it was not possible to arrive to the best solution and the computational time was three times the one required by eSS (see the corresponding convergence curves in Figure S.20) .

CVP	Solver	Best	Worst	Mean	CPU time(s)
PWL-v	eSS (FMINCON)	95.532410	93.673200	94.880798	454
	**eSS(SOLNP)**	**95.765699**	**93.787700**	**95.066655**	**404**
	**eSS(FSQP)**	**95.765699**	**93.670710**	**95.081565**	**504**
	DE	92.692226	90.472200	92.053025	5032
	hyb (DE+FMINCON)	95.728577	86.386021	92.178920	1474
	hyb (DE+SOLNP)	95.359738	92.300028	94.821770	1225
PWC	ess (FMINCON)	95.681258	95.428801	95.623987	6096
	ess (SOLNP)	95.658784	95.455716	95.524084	7900

Table S.15: **Summary of results achieved with global methods for the *S. cerevisiae* related example.** Two CVP schemes were tested for the sake of comparison. In general, solutions are better with PWL-v interpolation and the optimum is found in around 7 to 8 min. The dispersion of the solutions is lower with the PWC approximation confirming that multimodality increases when switching times are included as decision variables.

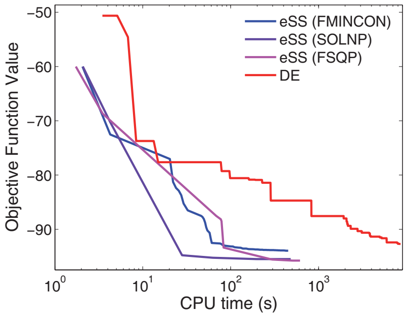

Figure S.20: **Comparison of convergence curves for the best runs in *S.cerevisiae***

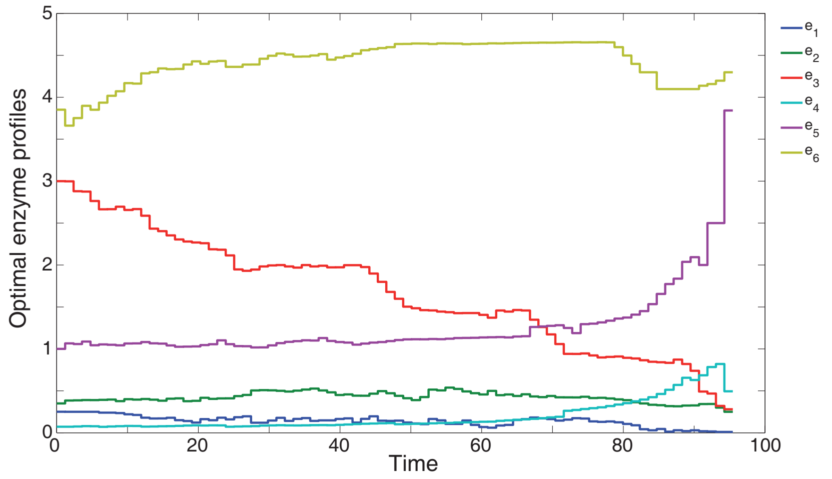

(a)

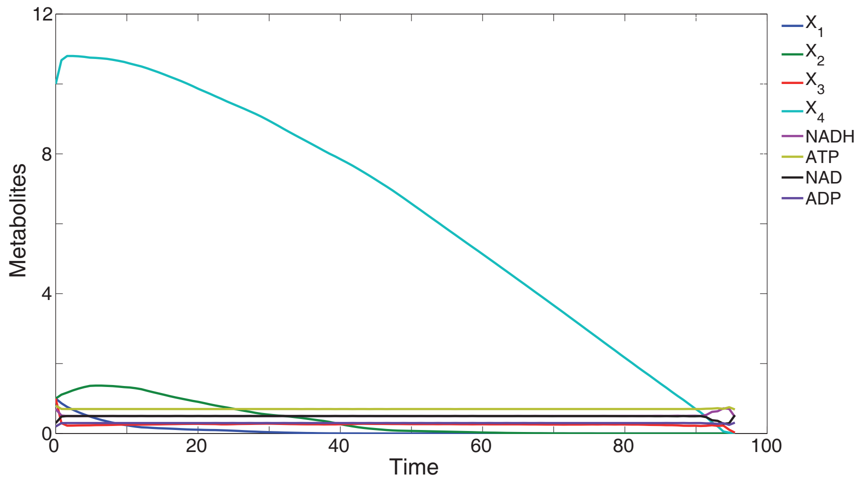

(b)

Figure S.21: **Optimal enzyme profiles with a PWC approximation and the corresponding states evolution in the central metabolism of *Saccharomyces cerevisiae* during diauxic shift**

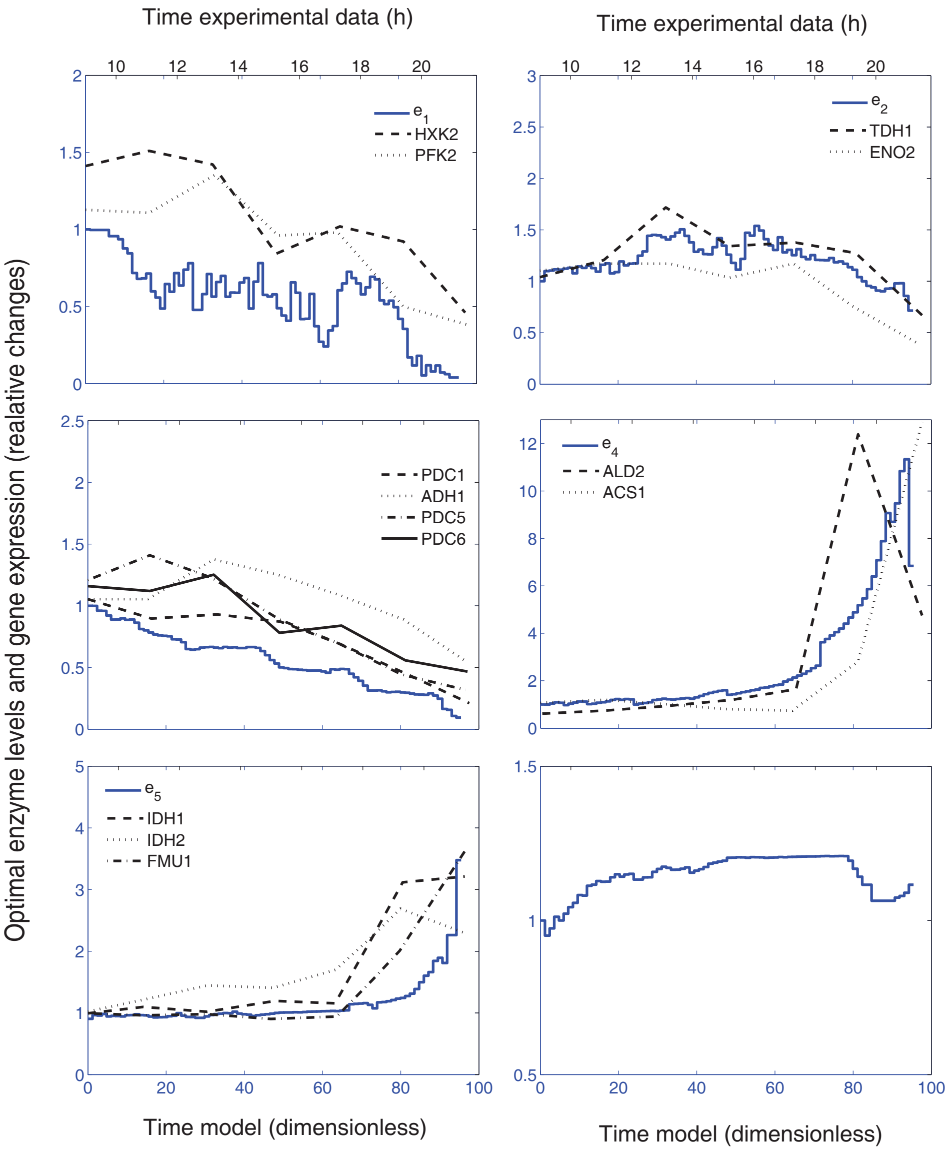

Figure S.22: **Comparison between experimental data and model predictions for PWC interpolation**. The enzyme profiles follow the general tendency of experimental data.

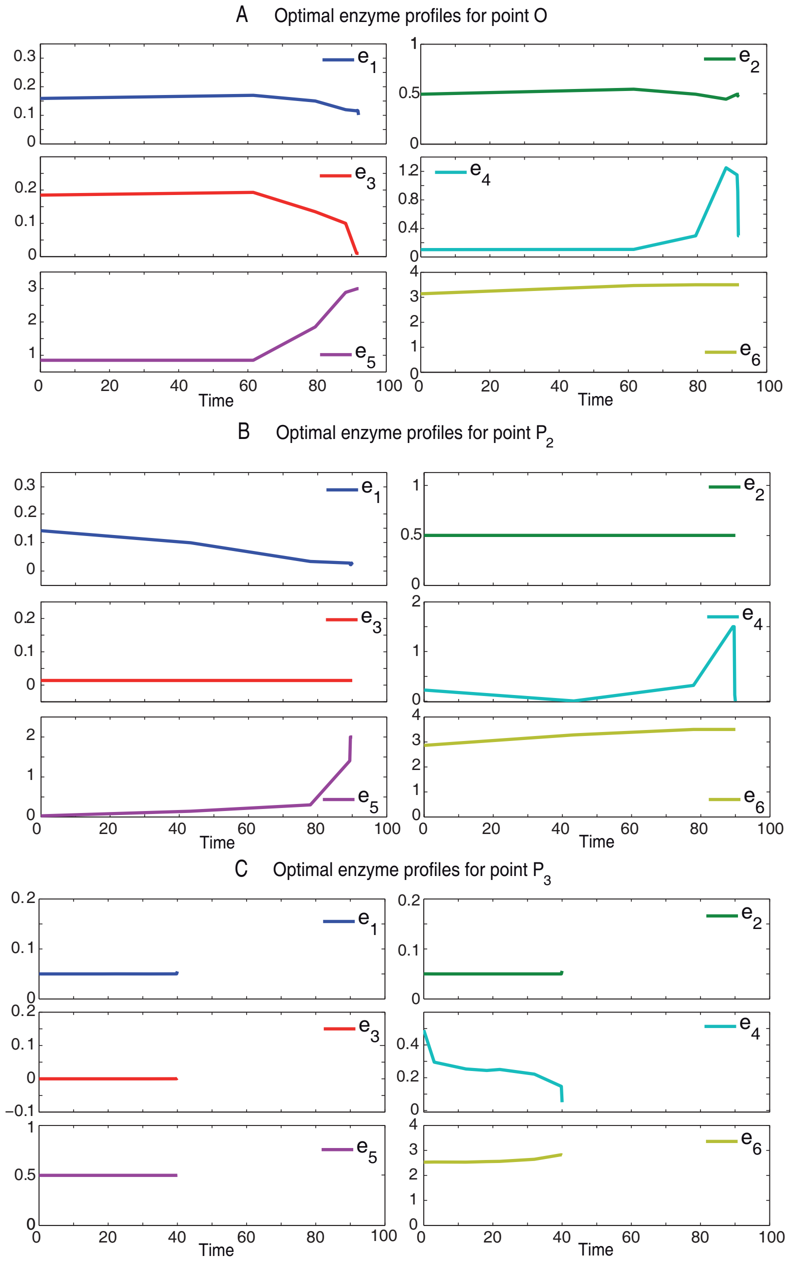

Figure S.23: **Optimal profiles (PWC interpolation) for the selected points of the Pareto-front (O,$\mathbf{P_2}$,$\mathbf{P_3}$) in SC**. In general, when enzyme consumption is reduced there is a reduction on the initial value of the enzymes and in the changes produced in their concentration during the process. Note that, for low consumptions (P_3) the enzymes keep their activation value almost constant except e_4.

References

1. Klipp E, Holzhütter HG, Heinrich R: **Reprogramming of the metabolic system by altering gene expression**. In *Proceedings of the 9th Int. Meeting on BioThermoKinetics* 2001:65–70.

2. Klipp E, Heinrich R, Holzhütter HG: **Prediction of temporal gene expression. Metabolic optimization by re-distribution of enzyme activities.** *European Journal of Biochemistry* 2002, **269**:5406–5413.

3. Oyarzún DA, Ingalls BP, Middleton RH, Kalamatianos D: **Sequential activation of metabolic pathways: a dynamic optimization approach**. *Bulletin of Mathematical Biology* 2009, **71**:1851–1872.

4. Bartl M, Li P, Schuster S: **Modelling the optimal timing in metabolic pathway activation-use of Pontryagin's Maximum Principle and role of the Golden section**. *Biosystems* 2010, **101**:67–77.

5. Bartl M, Kötzing M, Kaleta C, Schuster S, Li P: **Just-in-time activation of a glycolysis inspired metabolic network - solution with a dynamic optimization approach.** In *Proceedings 55nd International Scientific Colloquium. 2010. Ilmenau, Germany.* 2010.

6. Hairer E, Wanner G: *Solvind Ordinary Differential Equations II: Stiff and Differential Algebraic Problems.* Springer-Verlag 1996.

7. Fletcher R: *Practical Methods of Optimization.* John Wiley & Sons, Inc., New York, 2nd edition 1987.

8. Zhou JL, Tits AL, Lawrence CT: **User's Guide for FFSQP Version 3.7: A Fortran Code for Solving Optimization Programs, Possibly Minimax, with General Inequality Contraints and Linear Equality Constraints, Generating Feasible Iterates.** Tech. Rep. SRC-TR-92-107r5, Institute for systems research, University of Maryland 1997.

9. Ye Y: **Interior-point algorithms for global optimization**. *Ann. Oper. Res.* 1990, **25**:59–74.

10. Houska B, Ferreau HJ, Diehl M: **ACADO Toolkit - An Open-Source Framework for Automatic Control and Dynamic Optimization**. *Optimal Control Methods and Applications* 2011, **32**(3):298–312.

11. Runarsson TP, Yao X: **Stochastic ranking for constrained evolutionary optimization.** *IEEE Transactions on Evolutionary Computation* 2000, **564**:284–294.

12. Storn R, Price K: **Differential Evolution - a Simple and Efficient Heuristic for Global Optimization over Continuous Spaces**. *Journal of Global Optimization* 1997, **11**:341–359.

13. Balsa-Canto E, Vassiliadis VS, Banga JR: **Dynamic optimization of single- and multi-stage systems using a hybrid stochastic-deterministic method**. *Industrial & Engineering Chemistry Research* 2005, **44**(5):1514–1523.

14. Egea JA, Balsa-Canto E, Garcia MG, Banga JR: **Dynamic optimization of nonlinear processes with an enhanced scatter search method.** *Industrial & Engineering Chemistry Research* 2009, **48**(9):4388–4401.

15. Egea JA, Rodriguez-Fernandez M, Banga JR, Marti R: **Scatter search for chemical and bio-process optimization**. *Journal of Global Optimization* 2007, **37**(3):481–503.

16. Llorens M, Nuno J, Rodriguez Y, Melendez-Hevia E, Montero F: **Generalization of the theory of transition times in metabolic pathways: A geometrical approach.** *Biophysical Journal* 1999, **77**:22–36.

17. Oyarzún DA, Ingalls BP, Middleton RH, Kalamatianos D: **Optimal metabolic regulation by time variation of enzyme activities: a control theoretic approach.** In *Proceedings of Foundations of Systems Biology and Engineering* 2007:491–496.

18. Oyarzún DA: **A control-theoretic approach to dynamic optimization of metabolic networks**. *PhD thesis*, PhD thesis. National University of Ireland Maynooth, Ireland 2010.
